# Development and internal validation of machine learning–based models and external validation of existing risk scores for outcome prediction in patients with ischaemic stroke

**DOI:** 10.1093/ehjdh/ztad073

**Published:** 2023-11-22

**Authors:** Daniel Axford, Ferdous Sohel, Vida Abedi, Ye Zhu, Ramin Zand, Ebrahim Barkoudah, Troy Krupica, Kingsley Iheasirim, Umesh M Sharma, Sagar B Dugani, Paul Y Takahashi, Sumit Bhagra, Mohammad H Murad, Gustavo Saposnik, Mohammed Yousufuddin

**Affiliations:** Department of Information Technology, Mathematics and Statistics, College of Science, Health, Engineering and Education, Murdoch University, Murdoch, Australia; Department of Information Technology, Mathematics and Statistics, College of Science, Health, Engineering and Education, Murdoch University, Murdoch, Australia; Department of Public Health Science, Penn State College of Medicine, Hershey, PA, USA; Robert D. and Patricia E. Kern Centre for the Science of Healthcare Delivery, Mayo Clinic, Rochester, MN, USA; Neuroscience Institute, Geisinger Health System, 100 North Academy Ave, Danville, PA 17822, USA; Neuroscience Institute, The Pennsylvania State University, Hershey, PA 17033, USA; Internal Medicine/Hospital Medicine, Brigham and Women’s Hospital, Harvard University, Boston, MA, USA; Internal Medicine/Hospital Medicine, West Virginial University, Morgantown, WV, USA; Internal Medicine/Hospital Internal Medicine, Mayo Clinic Health System, Mankato, MN, USA; Hospital Internal Medicine, Mayo Clinic, Phoenix, AZ, USA; Hospital Internal Medicine, Mayo Clinic, Rochester, MN, USA; Community Internal Medicine, Mayo Clinic, Rochester, MN, USA; Endocrinology, Diabetes and Metabolism, Mayo Clinic Health System, Austin, MN, USA; Division of Public Health, Infectious Diseases, and Occupational Medicine, Mayo Clinic, Rochester, MN, USA; Stroke Outcomes and Decision Neuroscience Research Unit, Division of Neurology, Department of Medicine and Li Ka Shing Knowledge Institute, St.Michael’s Hospital, University of Toronto, Toronto, Ontario, Canada; Hospital Internal Medicine, Mayo Clinic Health System, 1000 1st Drive NW, Austin, MN 55912, USA

**Keywords:** Stroke, Mortality, Prediction models, Machine-based learning, Statistical

## Abstract

**Aims:**

We developed new machine learning (ML) models and externally validated existing statistical models [ischaemic stroke predictive risk score (iScore) and totalled health risks in vascular events (THRIVE) scores] for predicting the composite of recurrent stroke or all-cause mortality at 90 days and at 3 years after hospitalization for first acute ischaemic stroke (AIS).

**Methods and results:**

In adults hospitalized with AIS from January 2005 to November 2016, with follow-up until November 2019, we developed three ML models [random forest (RF), support vector machine (SVM), and extreme gradient boosting (XGBOOST)] and externally validated the iScore and THRIVE scores for predicting the composite outcomes after AIS hospitalization, using data from 721 patients and 90 potential predictor variables. At 90 days and 3 years, 11 and 34% of patients, respectively, reached the composite outcome. For the 90-day prediction, the area under the receiver operating characteristic curve (AUC) was 0.779 for RF, 0.771 for SVM, 0.772 for XGBOOST, 0.720 for iScore, and 0.664 for THRIVE. For 3-year prediction, the AUC was 0.743 for RF, 0.777 for SVM, 0.773 for XGBOOST, 0.710 for iScore, and 0.675 for THRIVE.

**Conclusion:**

The study provided three ML-based predictive models that achieved good discrimination and clinical usefulness in outcome prediction after AIS and broadened the application of the iScore and THRIVE scoring system for long-term outcome prediction. Our findings warrant comparative analyses of ML and existing statistical method–based risk prediction tools for outcome prediction after AIS in new data sets.

## Graphical Abstract

**Graphical Abstract:**
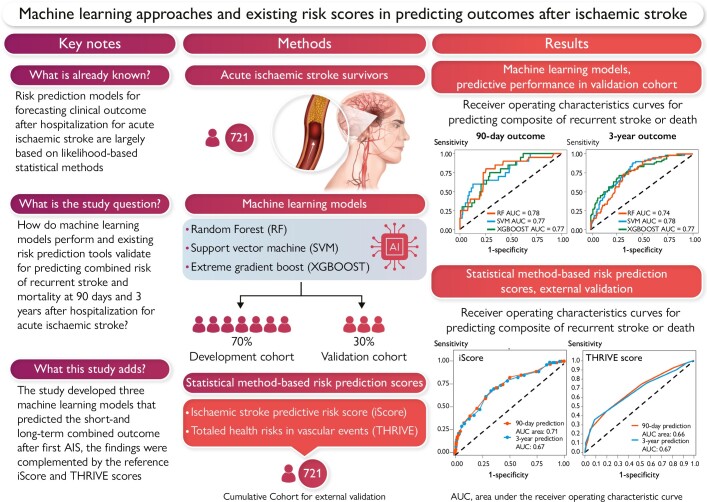

## Introduction

In the USA, stroke is one of the leading causes of hospitalization^1^ with high rates of stroke recurrence (15–18%) and mortality (29–48%) after 3 years, despite considerable advances in drug and device interventions.^2–4^ Accurate risk stratification of inpatients with an acute ischaemic stroke (AIS) is crucial for guiding targeted interventions and clinical decision-making.^5^ Evidence suggests that even stroke experts achieve <50% accuracy in predicting the clinical outcome after incident stroke.^6^ In previous studies largely based on conventional likelihood–based statistical (CS) methods, the investigators have reported individual predictors of recurrent stroke or mortality following initial stroke.^7^

Numerous risk prediction models, largely based on CS methods, have been developed for predicting short-term clinical outcomes to improve inpatient care and offer limited clinical utility.^8–12^ Acute ischaemic stroke is a complex and heterogenous condition with a wide range of variables that potentially determine the eventual outcome, especially in the long term. Currently available risk prediction models for AIS use a limited number of candidate predictors to compute the risk of outcome.^8–12^ These risk prediction models fail to account for a large number of variables^13^ and, therefore, performed poorly for outcome prediction when compared with machine learning (ML) models that were trained on a larger number of variables.^14^ However, artificial intelligence–based ML models provide unique analytic opportunities to integrate large-scale information and condense and select the most relevant predictors from a broader high-dimensional data set for predicting outcome but have been under-investigated in AIS.^15,16^ Currently, none of the prediction models for AIS outcome have demonstrated usefulness in informing clinical decision-making using statistical methods such as decision curve analysis (DCA).^17^ Therefore, novel risk prediction models with robust methodology that account for diverse predictor variables with improved performance relative to existing models are needed for the risk stratification of patients with AIS. Furthermore, the present ML models and statistical method–based scoring systems for outcome prediction after hospitalization for AIS have been limited by short-term follow-up and uncertain clinical applicability. Their utility in predicting recurrent stroke and mortality beyond 1 year is unknown.

Accordingly, we aimed, primarily, to develop, calibrate, and validate novel ML models, trained on a broader array of predictor variables than previously reported,^18^ to predict the composite of recurrent stroke or all-cause mortality at 90 days or 3 years in AIS survivors after hospital discharge. Our secondary aims were to externally validate the ischaemic stroke predictive risk score (iScore)^19^ and totalled health risks in vascular events (THRIVE) scores,^20^ two well-known and extensively validated prognostic models as exemplars for identifying patients at high risk for composite outcomes after AIS, and to assess the performance of each ML model and risk score for clinical utility across a range of thresholds.

## Methods

### Study design and population

In this retrospective study, we screened 1024 patients for eligibility and identified a final cohort of 721 consecutive patients who survived their first-ever AIS after index hospitalization at the Mayo Clinic, Rochester, MN, from 1 January 2005 to 30 November 2016. The *International Classification of Disease Clinical Modification Tenth Revision* (ICD-10-CM) codes were used to identify primary discharge diagnoses (see Supplementary material online, *Panel S1*). The patients were followed up for recurrent stroke or death, whichever occurred first, until 30 November 2019; if neither occurred, the patients were censored on that day. The following patients were excluded: 45 patients (4%) with non-stroke conditions, 99 patients (10%) with previous strokes not categorized as AIS, and 159 patients (16%) aged ≥ 90 years (the Institutional Review Board restricts data sharing to patients aged below 90 years). We followed the transparent reporting of a multivariable prediction model for individual prognosis or diagnosis (TRIPOD) guidelines^21^ for study design, conduct, and reporting. We also adhered to the guidelines for strengthening the reporting of observational studies in epidemiology (STROBE). The STROBE flow diagram for selection of steady heart is provided in Supplementary material online, *Figure S1*. Data were extracted and deidentified prior to the analysis. The details of the data extraction process have been published elsewhere.^22^ The Mayo Clinic Institutional Review Board approved the study and waived the requirement for informed consent. The deidentified data that support the results of the present study are available upon specific request from the corresponding author.

### Baseline characteristics

Stroke was diagnosed through a physical examination supported by brain and vascular imaging. The first-ever stroke was defined as a stroke occurring in a patient who had not experienced any prior stroke within a minimal look-back period of 9 years. For each patient, the severity of AIS on admission was assessed using the National Institutes of Health Stroke Scale (NIHSS) score.^23^ The NIHSS score ranges from 0 to 42, with higher scores indicating greater severity. Acute ischaemic stroke subtypes were classified into five categories^24^: (i) large artery atherosclerosis; (ii) cardioembolism; (iii) small vessel occlusion; (iv) other determined aetiology; and (v) undetermined aetiology. On the day of dismissal, basic activities of daily living, including bathing, dressing, grooming, toileting, hygienic measures, mobility, and eating, were assessed, and individually awarded scores of 1 (independent), 2 (requiring assistance), and 0 (dependent). Potential predictors were selected from multiple domains for model training based on a review of the literature, previous work, and expert opinion.^22,25–28^ Potential predictors were selected from multiple domains (see Supplementary material online, *Table S1*). The STROBE flow diagram shows that 14 patients (1.4%) had one or more missing data points. The missing values in the development and validation sets were imputed to prevent data leakage using the missForest R package based on the random forest (RF) method.^29^ Body mass index (BMI) was calculated by dividing weight in kilograms by the square of the height in metres. Categorical variables were encoded in numerically to facilitate the input data in the development data set.

### Outcomes

We chose composite events as the outcome measure for the following reasons: We reviewed published literature and found considerable debate over the use of individual vs. combined clinical endpoints.^30,31^ Recent improvements in the treatment of AIS have led to a decline in the incidence of relevant outcomes that can be used as clinical endpoints, especially when challenged with low event rates.^32^ It is generally conceived that the higher the gradient in importance and frequency across the components of the combined outcome, the greater the inherent challenges in interpreting the results.^33^ Previous studies have clearly demonstrated that the risk of death is substantially greater after a recurrent stroke than after the first stroke,^34^ despite a decrease in the risk of stroke recurrence.^35^ These findings may argue for using aggregated recurrent stroke and mortality as the primary endpoint in stroke research.^33^ Combining recurrent stroke and mortality allowed for an evaluation of the overall impact and consequences of the event or intervention on patient health. Therefore, we choose a composite of recurrent stroke or death from any cause (both outcomes are of equal importance to the patient) within 90 days or 3 years in patients who survived to discharge after hospitalization for first-ever AIS as the outcome.

### Machine learning–based approach

We followed four crucial steps to identify the best-performing risk prediction algorithm: (i) development and calibration of ML-based risk prediction models on a randomly generated development (*n* = 505; 70%) data set; (ii) external validation of the iScore and THRIVE scoring systems for the cumulative cohort (*n* = 721); and (iii) employment of DCA to assess the net benefit across wide-ranging thresholds for each model.

We built three independent ML-based models after assessing different ML methods. These models were trained in a development data set to predict binary composite outcomes. The models were based on RF,^36^ support vector machine (SVM),^37^ and extreme gradient boosting (XGBOOST)^36^ algorithms. The ML models were carefully selected according to their performance in predicting AIS outcomes in prior modelling studies.^38,39^ The relative advantages and disadvantages of the ML models are listed in Supplementary material online, *Table S2*. All ML models were constructed using 90 variables (see Supplementary material online, *Table S1*) and trained using 10-fold cross-validation, which was repeated 10 times to improve the prediction of composite outcomes. We constructed separate prediction models in the development data set using the top 10 variables that contributed the most to model prediction.

Further details on the model development, hyperparameter evaluation, and class imbalance are provided in the Supplementary material online, *Panel S2*. A schematic representation of the development process of ML algorithms is shown in Supplementary material online, *Figure S2*.

### Ascertainment of ischaemic stroke predictive risk score and totalled health risks in vascular events scores

External validation refers to the evaluation of a prediction model’s performance outside the original model development setting to determine its reproducibility and generalizability.^40^ The Supplementary material online, *Table S3* describes and Supplementary material online, *Table S4* scores the components of the iScore and THRIVE scoring systems.

The iScore is a point-based prognostic score that was originally developed using a conventional statistical approach to predict the mortality rates within 30 days and 1 year following AIS in patients hospitalized from 2003 to 2008.^19^ We used the 30-day and 1-year iScore versions to validate the 90-day and 3-year composite outcomes in our study population. The score was calculated for each patient using a risk score algorithm. The score was generated based on age, sex, stroke severity, risk factors (atrial fibrillation, heart failure, coronary artery disease, and current smoking status), comorbid conditions (cancer and renal dialysis), and blood glucose levels on admission. Because of data limitations, comorbid diabetes mellitus was substituted for an admission blood glucose level of >130 mg/dL. The NIHSS score was converted to the Canadian Neurological Society score to validate the iScore in our study population.

The THRIVE score is also a point-based risk prediction tool derived from logistic regression.^20^ The THRIVE scoring system was initially developed to determine outcomes after endovascular treatment for AIS and was subsequently externally validated in patients with general stroke.

Both iScore and THRIVE models were evaluated using the area under the receiver operating characteristic curve (AUC), calibrated using the estimated slope and intercept. The goodness of fit was assessed using the Hosmer–Lemeshow test. The Youden index was calculated based on sensitivity and specificity. The maximum Youden index value was identified, and the corresponding sensitivity and specificity values were reported. Additionally, the positive predictive value (PPV) and negative predictive value (NPV) were calculated using the prevalence of adverse outcomes in the study sample. Additionally, the iScore and THRIVE scores were directly compared to predict the 3-year composite outcome.

### Statistical analysis

Analyses were conducted using RStudio (R 4.0.516) for Windows, Stata (SE version 17.0; Stata Corp LLC, College Station, TX) and SAS statistical programs. We report the mean and standard deviation (SD) for normally distributed variables, the median and interquartile range (IQR) for non-normally distributed variables, and the number and proportion of categorical variables. Normally distributed data, non-normally distributed data, and categorical variables were compared using Student’s *t*-test, the Kruskal–Wallis test, and the Pearson *χ*^2^ test, respectively.

### Model performance metrics

The performance metrics of all ML models for binary outcomes were measured as AUC for discriminative ability, Brier score for overall model performance, goodness of fit for calibration, and DCA for net benefit decision-making using a recently recommended framework.^41^ The Brier score is very familiar to the research community for evaluating and comparing ML models.^42^ It is an accepted metric for evaluating probabilistic prediction, particularly in the context of clinical prediction models. The Brier score effectively assesses the calibration and accuracy of predicted probabilities, which is crucial in various medical applications. Furthermore, the Brier score facilitates direct model comparisons by quantifying the average scored difference between predicted probabilities and observed outcomes. We aimed to optimize hyperparameters based on the Brier score to enhance model’s abilities to provide an accurate and well-calibrated probability estimate. A lower Brier score and higher AUC represent better performance of ML model.

The discriminatory performance of each model was assessed using receiver operating characteristic (ROC) curve analysis in both the development and internal validation cohorts with all 90 variables and the top 10 most predictive variables. Differences in the AUC across the models were assessed using the DeLong test. Calibration plots were constructed for each model in both the development and validation cohorts with all 90 variables and the top 10 most predictive variables in each data set. The significance of the calibration plots was assessed using the Brier score [which ranges from 0 (best) to 1 (worst)]^43^ and Hosmer–Lemeshow^44^ goodness-of-fit test statistics (well calibrated if *P* > 0.05). Separate ROC curves, calibration plots, and decision analytic curves were generated in the development and validation data sets for the ML-based models and in the cumulative data set for the iScore and THRIVE scores.

The DCA is an approach to quantifying the utility of a prediction model for clinical decision-making.^17^ Decision curve analysis measures a ‘net benefit’ as a function of default strategies of treating all or treating none across a range of threshold probabilities.^45^ The net benefit complements the accuracy metrics of the model by incorporating the consequences of decisions made based on the prediction model. We assessed the net benefit of clinical utility across a range of thresholds for each model and the existing scoring system to ascertain whether the model should be used for clinical decision-making. In our analysis, we utilized the dcurves package in R to conduct the DCA. The net benefit derived from the DCA for all three ML models was computed using the 30% validation cohort. In contrast, the net benefit for the iScore and THRIVE was computed for the cumulative cohort.

First, we compared the results of the RF, SVM, and XGBOOST models in the development and internal validation data sets using the AUC, accuracy, Brier score, and Hosmer–Lemeshow test. Second, we compared the performance metrics for outcome prediction between study models to those derived from the validation and recalibration of the iScore and THRIVE scores in our study cohort.

We acknowledge that class imbalance is an important consideration in ML. However, in our study, we chose not to specifically address class imbalance because we wanted to evaluate the performance of our models in a real-world scenario where class imbalance is often present and not always addressed. By not explicitly handling the class imbalance, we aimed to have the models learn from the natural data distribution since it would be encountered in practice. This approach allows us to assess the models’ performance under real-world conditions and provides insights into their generalizability.

### Selection of the top 10 variables for the construction of parsimonious machine learning models

We selected the top 10 variables for the construction of parsimonious models for predicting outcomes from the following clinical and modelling perspectives: Stroke is a complex clinical syndrome with variability in outcome. Multivariable risk-adjusted models were superior to expert opinion for predicting stroke outcome.^6^ Variables in multiple domains improve the performance of a model derived from six clinical variables.^46^ The NIHSS score, which is the current gold standard for the assessment of disease severity, utilizes 13 features.^23^ In this study, we used features such as measures of basic activities of daily living, which are very important in the assessment of stroke recovery. Five variables may not be able to cover all the important domains of a condition as complex as AIS, whereas >10 variables become burdensome for bedside use; therefore, we choose to use 10 features for parsimonious model. From a modelling perspective, we chose to focus on the top 10 features to strike a balance between model complexity and interpretability. While other choices, such as selecting 5 or 15 features, could have been made, the selection of 10 features allowed us to capture a meaningful subset of highly important variables without overwhelming the model or sacrificing its interpretability.

### Complementary analysis

The THRIVE score was originally developed for predicting 90-day mortality in patients with AIS receiving thrombolytic therapy; subsequently, it exhibited broader utility and validation in other stroke types and for predicting in-hospital or 1-year mortality.^47–49^ Similarly, the iScore was initially developed to predict 3-month and 1-year mortality after AIS and subsequently validated at different time points.^50^ Although our primary objective was to assess whether the iScore or THRIVE scoring system can predict outcomes at 90 days and beyond 1 year (3 years in the present study) after index AIS, we constructed separate ROC curves to predict composite outcomes and mortality within 1 year of AIS to assess potential differences in discriminatory function. Additionally, we (i) validated and compared the iScore and THRIVE scores in a sensitivity analysis in a restricted 30% validation cohort for fairer evaluation against ML models and (ii) compared iScore and THRIVE scores for predicting a 90-day composite outcome.

The STROBE checklist is provided in Supplementary material online, *Table S5*.

## Results

### Study population

The median age in the study population was 75 years (IQR: 65–83 years); 49% of the patients were men, and 8% were non-white. During the follow-up, 80 (11%) and 246 (34%) patients experienced 90-day and 3-year outcomes, respectively. Baseline patient characteristics according to the study outcomes are presented in *Table 1*.

**Table 1:** Baseline characteristics of the study population by outcome

Variables	Cumulative cohort, *n* = 721	90-day composite of recurrent stroke and mortality			3-year composite of recurrent stroke or mortality		
		No (*n* = 641)	Yes (*n* = 80)	*P* value	No (*n* = 482)	Yes (*n* = 239)	*P* value
Age, median (IQR)	75.3 (65.0, 82.5)	74.8 (63.5, 82.2)	80.5 (74.4, 85.4)	<0.001	73.0 (61.6, 80.3)	81.0 (72.9, 85.3)	<0.001
Sex at birth, female	355 (49.2%)	328 (51.2%)	27 (33.8%)	0.003	249 (51.7%)	106 (44.4%)	0.065
Race, non-white	55 (7.6%)	47 (7.3%)	8 (10.0%)	0.397	35 (7.3%)	20 (8.4%)	0.598
BMI, median (IQR)	27 (24, 37)	27 (24, 32)	25.9 (22, 30)	0.061	28 (24, 32)	26 (23, 30)	<0.001
Current smoker	130 (18.0%)	124 (19.3%)	6 (7.5%)	0.009	94 (19.5%)	36 (15.1%)	0.144
LOS, median (IQR)	3.0 (2.0, 6.0)	3.0 (2.0, 5.0)	5.0 (2.8, 8.0)	<0.001	3.0 (2.0, 5.0)	4.0 (3.0, 7.0)	<0.001
Hyperlipidaemia	464 (64.4%)	424 (66.1%)	40 (50.0%)	0.004	336 (69.7%)	128 (53.6%)	<0.001
Hypertension	580 (80.4%)	517 (80.7%)	63 (78.8%)	0.685	379 (78.6%)	201 (84.1%)	0.081
Depression	138 (19.1%)	118 (18.4%)	20 (25.0%)	0.158	77 (16.0%)	61 (25.5%)	0.002
Diabetes mellitus	196 (27.2%)	179 (27.9%)	17 (21.2%)	0.206	128 (26.6%)	68 (28.5%)	0.59
Arthritis	190 (26.4%)	168 (26.2%)	22 (27.5%)	0.805	118 (24.5%)	72 (30.1%)	0.105
Cancer	47 (6.5%)	37 (5.8%)	10 (12.5%)	0.022	14 (2.9%)	33 (13.8%)	<0.001
Atrial fibrillation	212 (29.4%)	180 (28.1%)	32 (40.0%)	0.027	123(25.5%)	89 (37.2%)	0.001
Asthma	34 (4.7%)	31 (4.8%)	3 (3.8%)	0.666	25 (5.2%)	9 (3.8%)	0.397
CAD	205 (28.4%)	177 (27.6%)	28 (35.0%)	0.167	121 (25.1%)	84 (35.1%)	0.005
PAD	59 (8.2%)	51 (8.0%)	8 (10.0%)	0.529	27 (5.6%)	32 (13.4%)	<0.001
Substance use	49 (6.8%)	43 (6.7%)	6 (7.5%)	0.791	33 (6.8%)	16 (6.7%)	0.939
COPD	64 (8.9%)	53 (8.3%)	11 (13.8%)	0.104	42 (8.7%)	22 (9.2%)	0.827
Osteoporosis	74 (10.3%)	60 (9.4%)	14 (17.5%)	0.024	36 (7.5%)	38 (15.9%)	<0.001
CKD	87 (12.1%)	70 (10.9%)	17 (21.2%)	0.007	44 (9.1%)	43 (18.0%)	<0.001
Heart failure	80 (11.1%)	62 (9.7%)	18 (22.5%)	<0.001	33 (6.8%)	47 (19.7%)	<0.001
Valve disease	53 (7.4%)	45 (7.0%)	8 (10.0%)	0.336	34 (7.1%)	19 (7.9%)	0.664
Dementia	69 (9.6%)	50 (7.8%)	19 (23.8%)	<0.001	25 (5.2%)	44 (18.4%)	<0.001
Seizure	6 (0.8%)	5 (0.8%)	1 (1.2%)	0.663	4 (0.8%)	2 (0.8%)	0.992
Anaemia	76 (10.5%)	64 (10.0%)	12 (15.0%)	0.168	35 (7.3%)	41 (17.2%)	<0.001
Migraine	26 (3.6%)	24 (3.8%)	2 (2.5%)	0.572	20 (4.2%)	6 (2.5%)	0.264
OSA	49 (6.8%)	44 (6.9%)	5 (6.2%)	0.837	34 (7.1%)	15 (6.3%)	0.696
Parkinson’s disease	11 (1.5%)	8 (1.2%)	3 (3.8%)	0.085	3 (0.6%)	8 (3.3%)	0.005
Liver disease	9 (1.2%)	8 (1.2%)	1 (1.2%)	0.999	6 (1.2%)	3 (1.3%)	0.991
Hypothyroidism	63 (8.7%)	53 (8.3%)	10 (12.5%)	0.206	40 (8.3%)	23 (9.6%)	0.553
Other psychiatric conditions	1 (0.1%)	0 (0.0%)	1 (1.2%)	0.005	0 (0.0%)	1 (0.4%)	0.155
Unilateral symptoms	684 (94.9%)	608 (94.9%)	76 (95.0%)	0.955	458 (95.0%)	226 (94.6%)	0.792
MCA	489 (67.8%)	432 (67.4%)	57 (71.2%)	0.486	311 (64.5%)	178 (74.5%)	0.007
Anterior circulation	536 (74.3%)	475 (74.15)	61 (76.2%)	0.678	341 (70.7%)	195 (81.6%)	0.002
Aetiology of stroke				<0.001			<0.001
LAA	83 (11.5%)	75 (11.7%)	8 (10.0%)		56 (11.6%)	27 (11.3%)	
Cardioembolism	216 (30.0%)	182 (28.4%)	34 (42.5%)		127 (26.3%)	89 (37.2%)	
SVO	158	154	4 (5.0%)		127 (26.3%)	31 (13.0%)	
Undetermined	226 (31.3%)	194 (30.3%)	32 (40.0%)		143 (29.7%)	83 (34.7%)	
Determined	38 (5.3%)	36 (5.6%)	2 (2.5%)		29 (6.0%)	9 (3.8%)	
NIHSS score, median (IQR)	4.0 (2.0,8.0)	4.0 (2.0,7.0)	8.0 (4.0,16.0)	<0.001	4.0 (2.0,6.0)	6.0 (3.0,10.5)	<0.001
SBP, median (IQR)	148 (134, 166)	149 (135, 165)	140 (132, 170)	0.286	149 (135, 166)	146 (134, 164)	0.203
DBP, median (IQR)	77.3 (69, 86)	77 (70, 86)	76.0 (66, 86)	0.261	77 (7 087)	76 (67, 85)	0.219
Thrombolytics	71 (9.8%)	63 (9.8%)	8 (10.0%)	0.961	45 (9.3%)	26 (10.9%)	0.513
Embolectomy	3 (0.4%)	3 (0.5%)	0 (0.0%)	0.54	3 (0.6%)	0 (0.0%)	0.222
Aspirin	487 (67.5%)	449(70%)	38 (47.5%)	<0.001	340(70.5%)	147 (61.5%)	0.015
Clopidogrel	132(18.3%)	119(18.6%)	13 (16.2%)	0.614	95 (19.7%)	37 (15.5%)	0.167
Aggrenox	49 (6.8%)	48 (7.5%)	1 (1.2%)	0.037	37 (7.7%)	12 (5.0%)	0.182
Warfarin	218 (30.2%)	199 (31%)	19 (23.8%)	0.18	149 (30.9%)	69 (28.9%)	0.574
DOACs	16 (2.2%)	15 (2.3%)	1 (1.2%)	0.533	6 (1.2%)	10 (4.2%)	0.012
Statin	473 (65.6%)	438 (68.3%)	35 (43.8%)	<0.001	338(70.1%)	135 (56.5%)	<0.001
High-intensity statin	21 (2.9%)	20 (3.1%)	1 (1.2%)	0.348	15 (3.1%)	6 (2.5%)	0.651
ACEI/ARBs	301(41.7%)	274 (42.7%)	27 (33.8%)	0.124	200 (41.5%)	101 (42.3%)	0.844
Beta-blockers	357 (49.5%)	327 (51%)	30 (37.5%)	0.023	227 (47.1%)	130 (54.4%)	0.065
Calcium channel blocker	127(17.6%)	105(16.4%)	22 (27.5%)	0.014	73 (15.1%)	54 (22.6%)	0.013
HCTZ	226 (31.3%)	204 (31.8%)	22 (27.5%)	0.432	155 (32.2%)	71 (29.7%)	0.504
Oral diabetic drugs	83 (11.5%)	82 (12.8%)	1 (1.2%)	0.002	66 (13.7%)	17 (7.1%)	0.009
Insulin	82 (11.4%)	74 (11.5%)	8 (10.0%)	0.682	46 (9.5%)	36 (15.1%)	0.028
SSRI	97 (13.5%)	88 (13.7%)	9 (11.2%)	0.54	57 (11.8%)	40 (16.7%)	0.069
Antiepileptics	24 (3.3%)	23 (3.6%)	1 (1.3%)	0.29	17 (3.5%)	7 (3.0%)	0.702
Antiplatelets	579 (80.3%)	534 (83.3%)	45 (56.2%)	<0.001	403 (83.6%)	176 (73.6%)	0.002
All anticoagulant	234 (32.5%)	214 (33.4%)	20 (25.0%)	0.131	155 (32.2%)	79 (33.1%)	0.809
Antiplatelet or anticoagulant	684 (94.9%)	629 (98.1%)	55 (68.8%)	<0.001	473 (98.1%)	211 (88.3%)	<0.001
Any antihypertensive	547 (75.9%)	497 (77.5%)	50 (62.5%)	0.003	363 (75.3%)	184 (77%)	0.62
Hemiparesis	325 (45.1%)	270 (42.1%)	55 (68.8%)	<0.001	188 (39%)	137 (57.3%)	<0.001
hemisensory defect	76 (10.5%)	63 (9.8%)	13 (16.2%)	0.078	43 (8.9%)	33 (13.8%)	0.044
Hemineglect	42 (5.8%)	36 (5.6%)	6 (7.5%)	0.498	25 (5.2%)	17 (7.1%)	0.299
Impaired speech	327 (45.4%)	285 (44.5%)	42 (52.5%)	0.173	205 (42.5%)	122 (51%)	0.031
Hemianopia	82 (11.4%)	70 (10.9%)	12 (15.0%)	0.279	53 (11.0%)	29 (12.1%)	0.65
Gaze palsy	64 (8.9%)	51 (8.0%)	13 (16.2%)	0.014	33 (6.8%)	31 (13.0%)	0.006
Facial palsy	322 (44.7%)	281 (43.8%)	41 (51.2%)	0.209	206 (42.7%)	116 (48.5%)	0.141
Arm weakness	418 (58%)	355 (55.4%)	63 (78.8%)	<0.001	252 (52.3%)	166 (69.5%)	<0.001
Lower extremity weakness	360 (49.9%)	308 (48%)	52 (65.0%)	0.004	216 (44.8%)	144 (60.3%)	<0.001
Arm and lower extremity weakness	330 (45.8%)	280 (43.7%)	50 (62.5%)	0.001	196 (40.7%)	134 (56.1%)	<0.001
Dysarthria	238(33%)	209 (32.6%)	29 (36.2%)	0.513	163 (33.8%)	75 (31.4%)	0.512
Ataxia	107 (14.8%)	101 (15.8%)	6 (7.5%)	0.05	84 (17.4%)	23 (9.6%)	0.006
Gait imbalance	74 (10.3%)	73 (11.4%)	1 (1.2%)	0.005	61 (12.7%)	13 (5.4%)	0.003
Vertigo	28 (3.9%)	27 (4.2%)	1 (1.2%)	0.196	24 (5.0%)	4 (1.7%)	0.031
Coma	11 (1.5%)	5 (0.8%)	6 (7.5%)	<0.001	3 (0.6%)	8 (3.3%)	0.005
Visual symptoms	41 (5.7%)	39 (6.1%)	2 (2.5%)	0.199	33 (6.8%)	8 (3.4%)	0.058
Incoordination	44 (6.1%)	42 (6.6%)	2 (2.5%)	0.153	35 (7.3%)	9 (3.8%)	0.065
Headache	22 (3.1%)	21 (3.3%)	1 (1.2%)	0.32	20 (4.1%)	2 (0.8%)	0.015
Confusion	11 (1.5%)	10 (1.6%)	1 (1.2%)	0.831	6 (1.2%)	5 (2.1%)	0.382
Dressing				<0.001			<0.001
0	90 (12.5%)	49 (7.6%)	41 (51.2%)		24 (5.0%)	66 (27.6%)	
1	374 (51.9%)	360 (56.2%)	14 (17.5%)		310 (64.3%)	64 (26.8%)	
2	257 (35.6%)	232 (36.2%)	25 (31.2%)		148 (30.7%)	109 (45.6%)	
Grooming				<0.001			<0.001
0	79 (11.0%)	42 (6.6%)	37 (46.2%)		18 (3.7%)	61 (25.5%)	
1	389 (54%)	372 (58%)	17 (21.2%)		318 (66%)	71 (29.7%)	
2	253 (35.1%)	227 (35.4%)	26 (32.5%)		146 (30.3%)	107 (44.8%)	
Toileting				<0.001			<0.001
0	87 (12.1%)	50 (7.8%)	37 (46.2%)		26 (5.4%)	61 (25.5%)	
1	377 (52.3%)	363 (56.6%)	14 (17.5%)		311 (64.5%)	66 (27.6%)	
2	257 (35.6%)	228 (35.6%)	29 (36.2%)		145 (30.1%)	112 (46.9%)	
Eating				<0.001			<0.001
0	61 (8.5%)	30 (4.7%)	31 (38.8%)		14 (2.9%)	47 (19.7%)	
1	519 (72%)	489 (76.3%)	30 (37.5%)		396 (82.2%)	123 (51.5%)	
2	141 (19.6%)	122 (19%)	19 (23.8%)		72 (14.9%)	69 (28.9%)	
Hygiene maintenance				<0.001			<0.001
0	84 (11.7%)	46 (7.2%)	38 (47.5%)		21 (4.4%)	63 (26.4%)	
1	395 (54.8%)	381 (59.4%)	14 (17.5%)		323 (67%)	72 (30.1%)	
2	242 (33.6%)	214 (33.4%)	28 (35.0%)		138 (28.6%)	104 (43.5%)	
Bathing				<0.001			<0.001
0	91 (12.6%)	52 (8.1%)	39 (48.8%)		27 (5.6%)	64 (26.8%)	
1	364 (50.5%)	352 (54.9%)	12 (15.0%)		302 (62.7%)	62 (25.9%)	
2	266 (36.9%)	237 (37%)	29 (36.2%)		153 (31.7%)	113 (47.3%)	
Mobility				<0.001			<0.001
0	96 (13.3%)	58 (9.0%)	38 (47.5%)		32 (6.6%)	64 (26.8%)	
1	372 (51.6%)	358 (55.9%)	14 (17.5%)		305 (63.3%)	67 (28.0%)	
2	253 (35.1%)	225 (35.1%)	28 (35.0%)		145 (30.1%)	108 (45.2%)	

0, dependent; 1, independent; 2, required assistance; ACEI/ARBs, angiotensin-converting enzyme inhibitor/angiotensin type II receptor blocker; BMI, body mass index; CAD, coronary artery disease; CKD, chronic kidney disease; COPD, chronic obstructive pulmonary disease; DOACs, direct-acting oral anticoagulants; DBP, diastolic blood pressure; HCTZ, hydrochlorothiazide; IQR, interquartile range, LAA, large artery atherosclerosis; LOS, length of stay; MCA, middle cerebral artery territory; *n*, number of patients; NIHSS, National Institutes of Health Stroke Scale; OSA, obstructive sleep apnoea; PAD, peripheral artery disease; SBP, systolic blood pressure; SVO, small vessel occlusion; SSRI, selective serotonin receptor inhibition.

### Machine learning models

**Figure 1:**
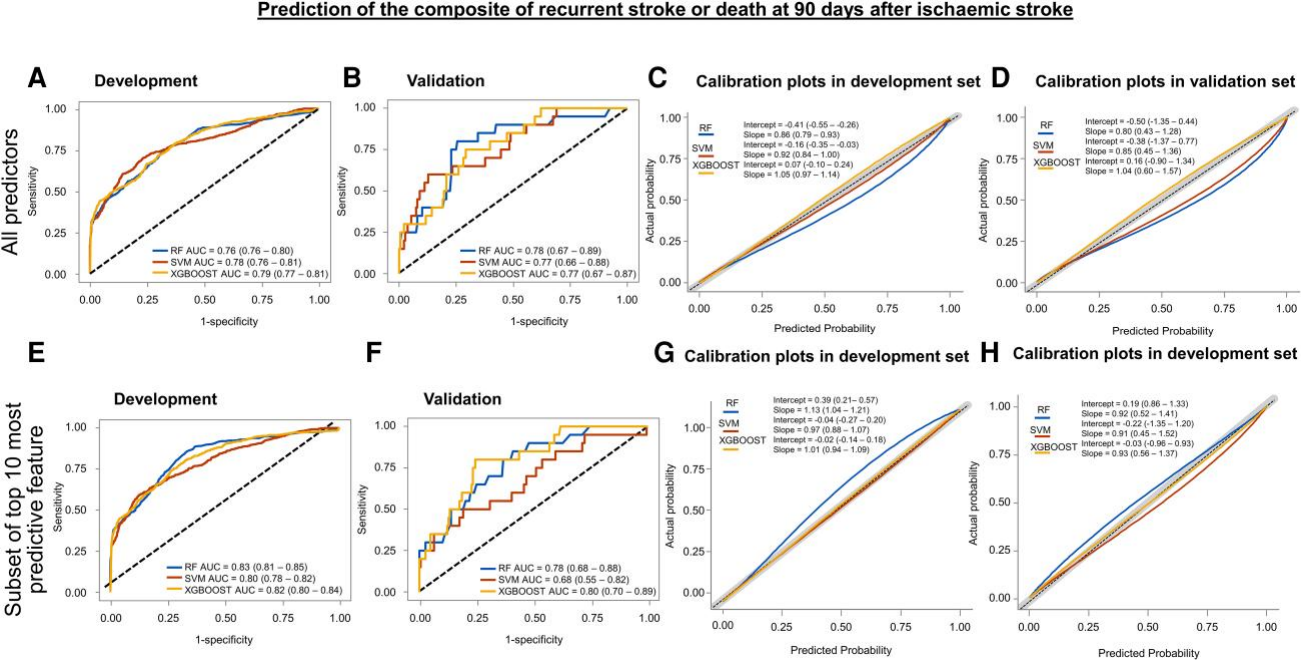
Receiver operating characteristic curves and calibration plots for 90-day outcome. Receiver operating characteristic curves for predicting composite or recurrent stroke or mortality at 90 days after hospitalization for acute ischaemic stroke are shown in the development (*A*) and internal validation (*B*) data sets stratified according to individual models using all the predictors. The corresponding calibration plots are depicted for the development (*C*) and internal validation (*D*) data sets. The lower panel illustrates receiver operating characteristic curves for each model [(*E*) development, (*F*) internal validation data sets] and calibration plots [(*G*) development, (*H*) internal validation data sets] for predicting 90-day outcome using the 10 most important predictors. AUC, area under the receiver operating characteristic curve; CI, confidence interval; RF, random forest; SVM, support vector machine; XGBOOST, extreme gradient boosting.

**Figure 2:**
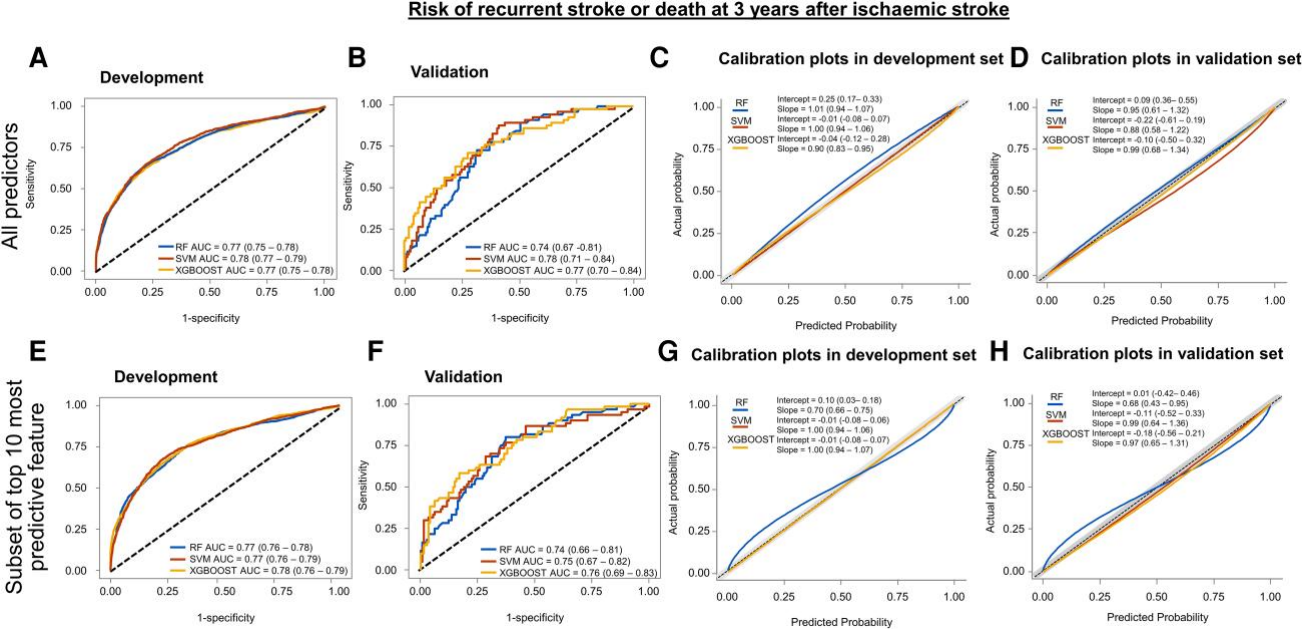
Receiver operating characteristic curves and calibration plots for 3-year outcome. Receiver operating characteristic curves for predicting composite or recurrent stroke or mortality at 3 years after hospitalization for acute ischaemic stroke are shown in the development (*A*) and internal validation (*B*) data sets stratified according to individual models using all the predictors. The corresponding calibration plots are depicted for the development (*C*) and internal validation (*D*) data sets. The lower panel illustrates receiver operating characteristic curves for each model [(*E*) development, (*F*) internal validation data sets] and calibration plots [(*G*) development, (*H*) internal validation data sets] for predicting 3-year outcome using the 10 most important predictors. AUC, area under the receiver operating characteristic curve; CI, confidence interval; RF, random forest; SVM, support vector machine; XGBOOST, extreme gradient boosting.

The ROC curves and calibration plots for each model for the 90-day and 3-year outcomes are shown in *Figures 1* and *2*, respectively. The full set of performance metrics based on the timescales of the outcomes is presented in *Tables 2* and *3*. Notably, all models achieved good discrimination and low Brier scores (indicators of the models’ performance). Additional details of the models are provided in Supplementary material online, *Table S6*. The TRIPOD checklist is provided in Supplementary material online, *Table S7*.

**Table 2:** Performance metrics of machine learning models for predicting composite of recurrent stroke or mortality at 90 days and 3 years after ischaemic stroke

Prediction	Features	Model	Development set, *n* = 505 (70%)			Validation set, *n* = 216 (30%)		
			AUC (95% CI)	Brier score	Hosmer–Lemeshow, *P*	AUC (95% CI)	Brier score	Hosmer–Lemeshow, *P*
90-day composite outcome	All 90 features	RF	0.782 (0.760–0.803)	0.079	0.915	0.779 (0.671–0.887)	0.072	0.692
		SVM	0.784 (0.762–0.806)	0.077	0.999	0.771 (0.660–0.882)	0.074	0.929
		XGBOOST	0.789 (0.768–0.810)	0.077	0.999	0.772 (0.674–0.869)	0.072	0.999
	Top 10 identified features	RF	0.828 (0.810–0.847)	0.076	0.944	0.777 (0.765–0.878)	0.072	0.999
		SVM	0.799 (0.778–0.819)	0.077	0.999	0.682 (0.547–0.817)	0.074	0.997
		XGBOOST	0.819 (0.800–0.838)	0.075	0.999	0.799 (0.704–0.894)	0.072	0.999
3-year composite outcome	All 90 features	RF	0.768 (0.754–0.782)	0.181	0.989	0.743 (0.674–0.811)	0.176	0.999
		SVM	0.779 (0.766–0.793)	0.173	0.999	0.777 (0.711–0.843)	0.171	0.958
		XGBOOST	0.765 (0.751–0.779)	0.177	0.999	0.773 (0.702–0.844)	0.161	0.999
	Top 10 identified features	RF	0.770 (0.756–0.784)	0.178	0.944	0.736 (0.663–0.839)	0.180	0.754
		SVM	0.773 (0.759–0.789)	0.175	0.999	0.746 (0.670–0.823)	0.171	0.999
		XGBOOST	0.776 (0.763–0.790)	0.174	0.999	0.763 (0.692–0.835)	0.166	0.999

AUC, area under the receiver operating characteristic curve; CI, confidence interval; RF, random forest; SVM, support vector machine; XGBOOST, extreme gradient boost.

**Table 3:** Performance metrics of ischaemic stroke predictive risk score and totalled health risks in vascular events score for predicting composite outcome at 90 days and 3 years after ischaemic stroke in cumulative cohort

Prediction	Model	Features	AUC (95% CI)	Cut-off	Sensitivity	Specificity	Youden’s index	PPV	NPV	Hosmer–Lemeshow, *P*
90-day composite outcome	iScore	8	0.715 (0.654–0.777)	70	0.700	0.647	0.347	0.199	0.945	0.081
	THRIVE	10	0.664 (0.595–0.731)	5	0.350	0.913	0.263	0.333	0.918	0.117
3-year composite outcome	iScore	10	0.671 (0.636–0.721)	40	0.657	0.622	0.279	0.463	0.785	0.124
	THRIVE	10	0.675 (0.663–0.716)	4	0.460	0.790	0.251	0.521	0.747	0.282

AUC, area under the receiver operating characteristic curve; CI, confidence interval; iScore, ischaemic stroke predictive risk score; THRIVE, totalled health risks in vascular events.

### Models with full set of predictors

#### Ninety-day prediction

The differences between the discriminatory performance of the RF [0.779, 95% confidence interval (CI) 0.671–0.887], SVM (0.771, 95% CI 0.660–0.882), and XGBOOST (0.772, 95% CI 0.674–0.869) models were not statistically significant in the validation data set.

#### Three-year prediction

The discriminatory performances of the RF (0.743, 95% CI 0.674–0.811), SVM (0.777, 95% CI 0.711–0.843), and XGBOOST (0.773, 95% CI 0.702–0.844) models were comparable in the validation data set.

### Top 10 most important variables

**Figure 3:**
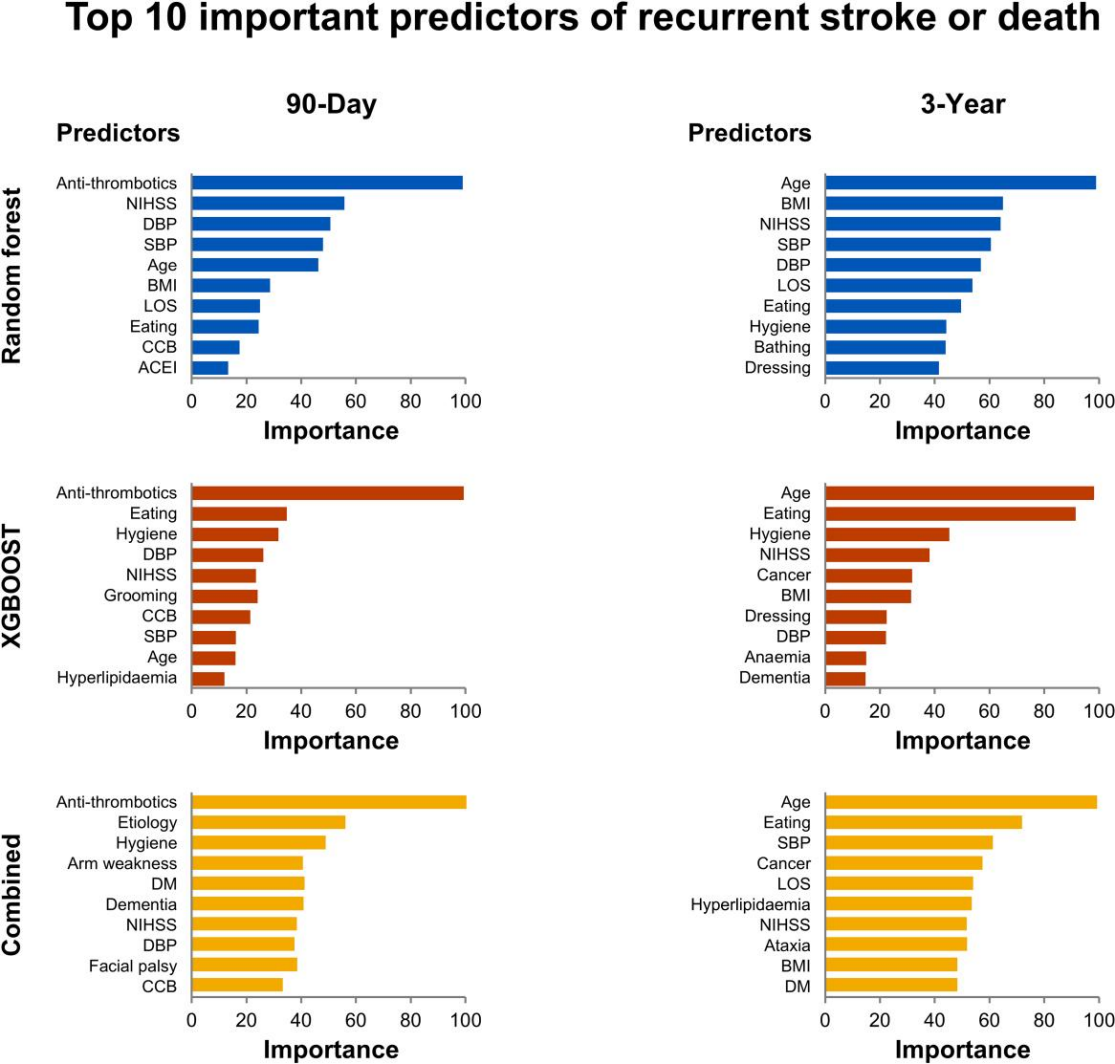
Top 10 important predictors for the composite outcome identified by random forest, extreme gradient boosting, and the average of all models. The bars represent the relative contribution of variables in predicting the clinical outcome. All models are consistent in identifying exposure/no exposure to antithrombotic drugs as the most important predictor for 90-day clinical outcome. Similarly, all models are consistent in identifying age as the most important predictor for 3-year clinical outcome. ACEI, angiotensin-converting enzyme inhibitor or angiotensin type II receptor blocker; BMI, body mass index; CCB, calcium channel blocker; DBP, diastolic blood pressure; DM, diabetes mellitus; LOS, length of stay; NIHSS, National Institutes of Health Stroke Scale; SBP, systolic blood pressure; XGBOOST, extreme gradient boosting.

The top 10 most important variables selected by each model from a total of 90 variables for predicting the composite outcome and the average of the two models are shown in *Figure 3*. The 10 important variables were derived solely from the training data set during the cross-validation process. All models consistently selected the variables ‘no antithrombotic drugs at discharge’ and ‘advanced age’ as important for predicting the 90-day and 3-year outcomes, respectively.

#### Ninety-day prediction

All models showed comparable performances; the RF, SVM, and XGBOOST models exhibited AUCs of 0.777 (95% CI 0.765–0.878), 0.682 (95% CI 0.547–0.817), and 0.799 (95% CI 0.704–0.894), respectively, in the validation set. The AUC achieved using 90 variables for the 90-day composite outcome was not greater than that achieved using the top 10 variables. Of the top 10 variables, no antithrombotic drugs at discharge, dependency on basic activities, NIHSS score, or prescription of calcium channel blockers at discharge were shared across the ML models. All three models selected antithrombotic medications at discharge as the most important variable.

#### Three-year prediction

The three ML models exhibited comparable performances: RF, SVM, and XGBOOST models showed AUC of 0.736 (95% CI 0.547–0.818), 0.746 (95% CI 0.670–0.823), and 0.763 (95% CI 0.692–0.835), respectively, in the validation set. The AUC achieved using 90 variables for the 3-year composite outcome was not greater than that achieved using the top 10 variables. Advanced age, dependency on basic activities, NIHSS score, and calcium channel blocker use at discharge were shared between the RF and XGBOOST models. Both the RF and XGBOOST models selected age as the most important factor.

### Validation of the ischaemic stroke predictive risk score and totalled health risks in vascular events scores

#### Ninety-day prediction

**Figure 4:**
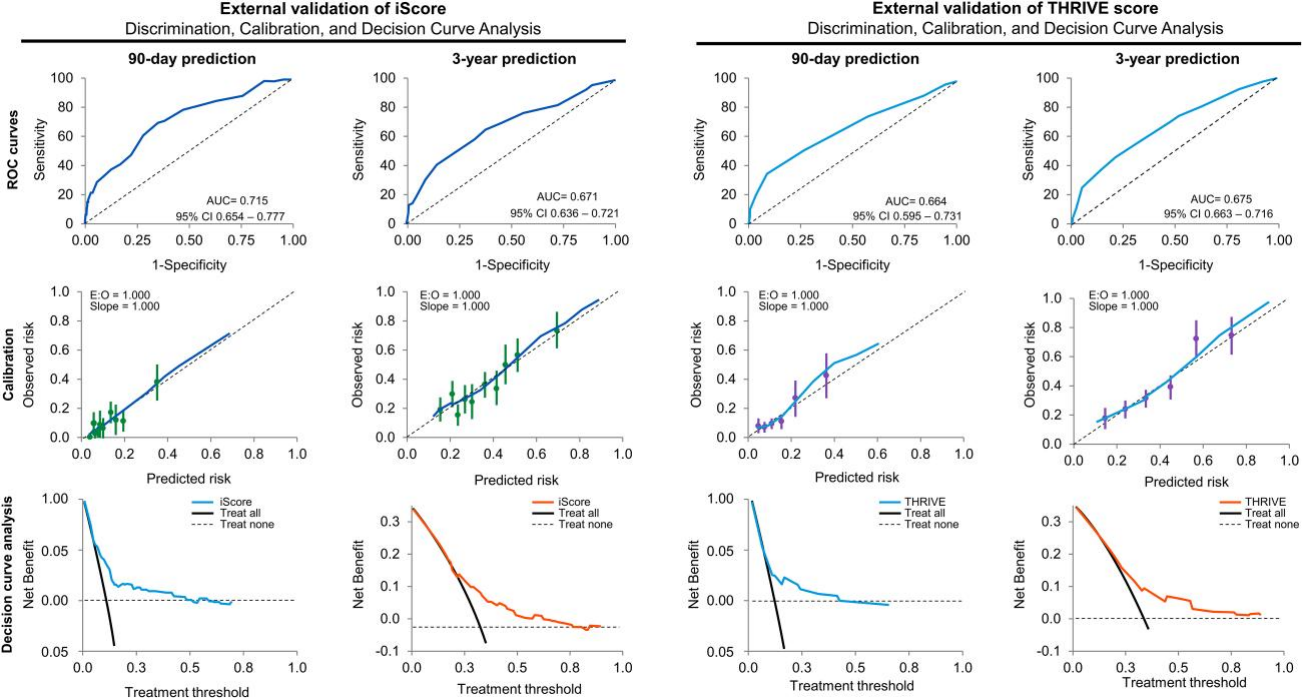
External validation with generation of receiver operating characteristic curves, calibration, and decision analysis curves of the ischaemic stroke predictive risk score and totalled health risks in vascular events scoring systems. The left two panels represent external validation of the ischaemic stroke predictive risk score with construction of receiver operating characteristic curves, recalibration plots, and decision curve analysis for 90-day and 3-year outcome prediction. Similarly, the right two panels represent the external validation of the totalled health risks in vascular events score with the generation of receiver operating characteristic curves, recalibration plots, and decision curve analysis for 90-day and 3-year outcome prediction.

The iScore achieved an AUC of 0.715 (95% CI 0.654–0.777) and good calibration (Hosmer–Lemeshow test, *P* = 0.081; *Figure 4*). At a cut-off point of 70, the iScore exhibited 70% sensitivity, 65% specificity, 20% PPV, and 95% NPV for the 90-day outcome prediction. The THRIVE score achieved a modest AUC of 0.664 (95% CI 0.595–0.731) and good calibration (Hosmer–Lemeshow test, *P* = 0.117). At a cut-off point of 5, the THRIVE score exhibited 35% sensitivity, 91% specificity, 33% PPV, and 92% NPV for the 90-day outcome prediction.

#### Three-year prediction

The iScore achieved an AUC of 0.671 (95% CI 0.636–0.721) and good calibration (Hosmer–Lemeshow test, *P* = 0.124). At a cut-off point of 40, the iScore exhibited 66% sensitivity, 62% specificity, 46% PPV, and 78% NPV for the 3-year outcome prediction. Similarly, the THRIVE score achieved an AUC of 0.675 (95% CI 0.663–0.716), with good calibration (Hosmer–Lemeshow test, *P* = 0.282). At a cut-off point of 4, the THRIVE score exhibited 46% sensitivity, 79% specificity, 52% PPV, and 75% NPV for the 3-year outcome prediction. The results of the direct comparison of the iScore (AUC 0.679; 95% CI 0.636–0.722) and THRIVE score (AUC 0.676; 95% CI 0.634–0.717) for predicting the 3-year outcome demonstrated no significant difference (DeLong test 0.886), as shown in Supplementary material online, *Figure S3* and *Table S8*.

### Decision curve analysis

**Figure 5:**
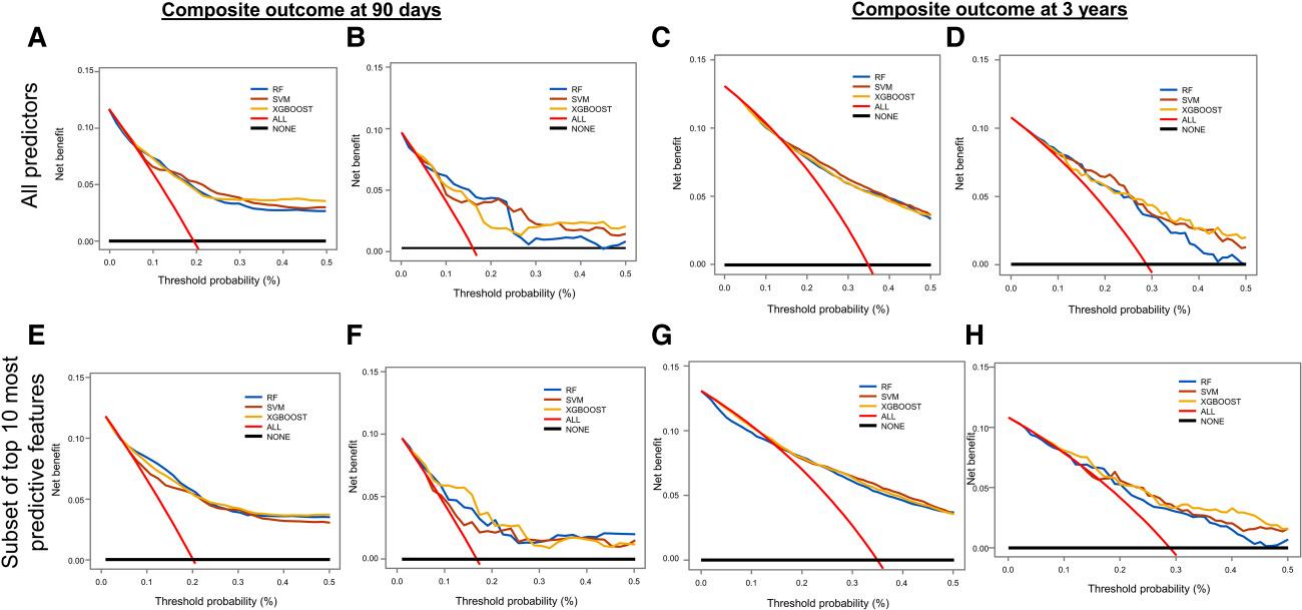
Decision curve analysis plots for each study model with all predictors are shown at 90 days in the development (*A*) and the internal validation sets (*B*) and at 3 years in the development (*C*) and internal validation sets (*D*). Similarly, decision curve analysis plots for each study model with the top 10 most important predictors are shown at 90 days in the development set (*E*) and internal validation set (*F*) and at 3 years in the development (*G*) and internal validation sets (*H*). RF, random forest; SVM, support vector machine; XGBOOST, extreme gradient boosting.

We plotted the net benefit on the *y*-axis and threshold probability (preferences) on the *x*-axis.^45^ Both the iScore and THRIVE scores provided comparable net benefits for clinical utility across a range of thresholds in the cumulative cohort for both the 90-day and 3-year outcome predictions (*Figure 4*). Random forest, SVM, and XGBOOST consistently showed net benefits across a wide range of thresholds for outcome prediction in both the development and validation data sets regardless of whether all 90 variables or the 10 most predictive variables were used to construct the plots (*Figure 5*).

### Comparative performances

The RF, SVM, and XGBOOST models and the iScore and THRIVE scoring systems demonstrated a good fit according to the Hosmer–Lemeshow test, with a *P* value consistently higher than 0.05. All ML models achieved numerically higher AUCs, greater accuracy, and lower Brier scores in predicting the 90-day than the 3-year composite outcomes after AIS. The AUCs for predicting both the 90-day and 3-year composite outcomes after AIS with all 90 or the top 10 most important variables were comparable across ML models in both the development and validation sets. The results of ML models were not statistically compared with the results of iScore and THRIVE scores because the validation of the ML models in 30% of the data set with the external validation of existing models in the cumulative cohort post challenges.

### Complementary analysis

The discriminatory performance of the iScore and THRIVE scoring systems for predicting 1-year composite events (iScore: AUC 0.680, 95% CI 0.631–0.730; THRIVE: AUC 0.663, 95% CI 0.609–0.717; DeLong *P* value 0.494) or 1-year mortality alone (iScore: AUC 0.733, 95% CI 0.682–0.785; THRIVE: AUC 0.713, 95% CI 0.656–0.770; DeLong 0.432) was not different from AUC for the 3-year prediction of composite events (see Supplementary material online, *Figure S4* and *Table S9*). Additional validation of the iScore and THRIVE scores in the 30% validation cohort yielded findings comparable to those of the primary analysis (iScore: AUC 0.679, 95% CI 0.636–0.721; THRIVE: AUC 0.676, 95% CI 0.634–0.717; DeLong 0.886), as shown in Supplementary material online, *Figure S5* and *Table S10*. We presented the sensitivity and specificity measures of ML models for outcome protection in Supplementary material online, *Table S11*. Finally, for 90-day composite outcome prediction, iScore outperformed THRIVE score (AUC 0.715 vs. 0.655; DeLong test *P* = 0.023) as shown in Supplementary material online, *Figure S6* and *Table S12*.

## Discussion

In this retrospective study, we assessed the performance and clinical usefulness of three ML models and validated the iScore and THRIVE score in predicting the 90-day or 3-year composite outcome of recurrent stroke or all-cause mortality after AIS. The important contributions of this study are as follows. In hospitalized AIS patients who survived to discharge, each of the three ML models, which were carefully developed using 90 predictor variables of high diversity, achieved similar performance in predicting the composite outcome at both 90 days and 3 years after AIS and similar usefulness for informing clinical decision-making. Additionally, the top 10 predictor-based parsimonious ML models retained the performance and clinical utility of the initial 90-variable-based models. Each study model demonstrated good discrimination and calibration in predicting the outcome over 90 days and 3 years. In this study, the utility of the iScore and THRIVE scoring systems was expanded for predicting recurrent stroke, predictive performance was extended beyond 1 year of follow-up, and clinical decision-making was improved through the construction of DCA curves.

The existing ML algorithms were modestly successful with a focus on functional outcomes and short-term follow-up (30 days to 1 year); they are insufficient for guiding routine clinical decision-making.^51,52^ None of the previous ML models examined the outcome beyond 1 year or systematically assessed their clinical usefulness using DCA.^51,52^ Conversely, the models built in this study have several advantages: The patients were well characterized with near-complete data collection and follow-up with only 0.1% missing values (which were imputed), the models were trained on manually abstracted large-scale granular data (total of 90 variables) from multiple domains to avoid artificial intelligence bias,^53^ and predictor variables such as those related to daily living activities not often used in previous studies were employed in model development.^54^

To avoid overfitting and to enhance generalizability, we developed and validated a separate set of pragmatic models for outcome prediction using the top 10 predictors from the original set of 90 variables with no loss of performance or clinical utility. We observed a predominance of stroke-associated variables contributing to the 90-day prediction (no antithrombotic medication use, stroke aetiology, arm weakness, dependency on daily activity, NIHSS score, and facial palsy), whereas demographics and comorbidities (age, dyslipidaemia, diabetes mellitus, cancer, BMI, and systolic blood pressure) prevailed in the 3-year prediction. Additionally, dependency in daily activities and NIHSS score were predictors for both the short- and long-term outcomes. Assessment of the directionality of variables indicated that small vessel occlusion stroke subtype (lacunar),^55^ increasing mean systolic blood pressure,^56^ hyperlipidaemia,^57^ and increasing BMI^58^ were inversely related to the 3-year composite outcome, consistent with previous reports.^7,22,59–63^

The performance characteristics of the ML models for risk prediction were similar in our study, consistent with several recent reports, including a meta-analysis of 71 studies.^18,51^ One potential explanation of these findings is the use of *a priori* variables as input features.^64^ The results might be different when variables are directly captured from large electronic databases. The study provides valuable insights into the performance metrics between the iScore and THRIVE score in predicting the composite of recurrent stroke or all-cause mortality. Although both the iScore and THRIVE scores were comparable in discriminatory performance, the THRIVE score demonstrated a lower sensitivity, allowing physicians to identify only 35 and 46% of the patients with AIS with composite outcomes at 90 days and 3 years, respectively, as opposed to the iScore, which allowed the identification of 70 and 66% of patients with composite outcomes at 90 days and 3 years, respectively. These findings suggest that using the THRIVE scores is likely to miss a portion of patients with unfavourable outcomes, supporting the findings of a previous report.^65^ Notably, the iScore and THRIVE scores consistently showed a net benefit across a range of thresholds in DCA, a finding not explored in previous validation studies.

The study was not intended to generate a point-based scoring system for risk calculation at the bedside, but, instead, to assess the performance of ML models in comparison to existing validated risk prediction tools. We conceived that ML models are complimentary to statistical-based models in providing meaningful insights and may not be mutually exclusive.^66^

### Clinical implications

Physicians are often faced with the difficult task of estimating a prognosis early after AIS to assist in decision-making, patient and family counselling, and resource allocation, but clinical expertise and knowledge of individual prognostic indicators are often insufficient for predicting clinical outcomes after AIS.^6^ Although both the iScore and THRIVE scores offer effective risk stratification after AIS, they have some limitations. Our advanced artificial intelligence–based ML models, equipped with improved decision curve analytics, provided enhanced accuracy and potentially scalable integration into workflows for informing clinical decision-making for short- and long-term follow-up in patients with AIS. Compared with statistical method–based risk prediction tools, ML models can be self-trained with additional data in the future to improve the current results and are not necessarily simple given the context of increasingly wider use of built-in automation of electronic medical records

### Research implications

There exists considerable uncertainty and confusion regarding artificial intelligence algorithms for prognostication, particularly among practising physicians, as opposed to the relatively small percentage of academics or physicians with interest in research. The overarching goal of our artificial intelligence research is making these algorithms more accessible to practising physicians. Therefore, in this study, we opted for iScore and THRIVE scoring systems, given their broad external validity as baseline models that are more familiar to practising physicians. We identified many opportunities and challenges for future research into the use of ML in outcome prediction, including the following: (i) advancing the design and innovation of the ML models to further improve their predictive performance; (ii) assessing the interoperability and generalizability of the models; (iii) testing the ability of the models to integrate into electronic health records for routine clinical use; and (iv) determining whether the models improve the clinical outcomes of individualized care and decision-making and reduce costs.

### Strengths and limitations

Our study had some limitations. Comparing the internal evaluation of ML models to the external validation of existing scores (iScore and THRIVE scores) presents a challenge in directly establishing the superiority of one approach over the other. Therefore, we did not compare ML models with the existing models. We applied novel ML strategies to data obtained from a single academic centre in predominantly Caucasian population involving adults aged <90 years; this design limited the generalizability of the study, and thus, external validation in an independent data set, especially one more inclusive of minority populations, is warranted. However, the study design minimizes the spectrum bias associated with large heterogeneous pooled data from multiple nations used in some studies, which limits model calibration and potential utility.^54^ Furthermore, the modest sample size precluded subgroup and sensitivity analyses. Although we included 90 variables, we cannot rule out the possibility of unmeasured confounders or misclassification because our data were extracted from electronic medical records. We evaluated the composite of recurrent stroke or mortality as the composite outcome because of low event rates and other reasons described above. The results may be different if components of the composite outcome are evaluated separately. This study also had several strengths. Comparing the internal evaluation of ML models to the external validation of existing scores (iScore and THRIVE scores) presents a challenge in directly establishing the superiority of one approach over the other. To enable equitable comparison, we generated new ROC curves for both the iScore and THRIVE scoring systems to predict 3-year composite outcomes in 30% validation data set. Notably, we found no discernable variations in predictive performance when compared with the results from the overall cohort. The study adhered to the TRIPOD reporting guidelines. We externally validated the previously identified risk prediction models, the iScore and the THRIVE scores, for reference to develop and validate ML models, extending their prognostic value to 3 years. Receiver operating characteristic curves, calibration plots, and decision analysis curves were created for each model for visual verification.

## Conclusions

In this retrospective study, three artificial intelligence–based ML models were developed to predict the short- and long-term combined risk of recurrent stroke or all-cause death after the first AIS. The ML models, iScore, and THRIVE score systems consistently demonstrated good discrimination and clinical utility in predicting the outcome across 90 days and 3 years after AIS. The utility of the iScore and THRIVE score has expanded to include recurrent stroke and extended prediction outcomes from 1 to 3 years after AIS. The ML models have the advantages of further improvement in the results by incorporating additional complex data in the future, self-training, automation, and ease of implementation in the workflow. Before wider clinical implementation, further research is needed to establish their generalizability in a larger and more heterogeneous patient population to ensure that predictive performance and clinical utility are preserved.

## Supplementary Material

ztad073_Supplementary_Data

## Data Availability

Anonymized data to support the results of this study will be made available upon request of qualified investigators.
