# Supplementary material for: Development and internal validation of machine learning–based models and external validation of existing risk scores for outcome prediction in patients with ischaemic stroke
Source: Eur Heart J Digit Health. 2023 Nov 22;5(2):109–22. doi: 10.1093/ehjdh/ztad073 (PMC10944684; doi:10.1093/ehjdh/ztad073)
Supplement: ztad073_Supplementary_Data [file ztad073_supplementary_data.pdf]

SUPPLEMENT

Development and internal validation of machine learning-based models and external validation of existing risk scores for outcome prediction in patients with ischemic stroke

Page no.

Supplemental Panel 1. International Classification of Disease Clinical Modification Tenth Revision ……………………..2

Supplemental Table 1. Full set of variables from multiple domains for training of machine learning models……………………………………………………………..3

Supplemental Table 2. Description of machine learning models……………………………………….........................4

Supplemental Table 3. Description iScore and THRIVE risk prediction tools……………………...........................5

Supplemental Table 4. Original iScore and THRIVE point-based prediction scores for validation and recalibration in the study cohort……………………………………………………………………………….6

Supplemental Figure 1. STROBE flow-diagram…………………………………………………………………………………….7

Supplemental Figure 2. Schematic representation of the process of the development of machine learning algorithms: datasets, methods of development, and cross-validation……………………8

Supplemental Table 5. STROBE checklist…………………………………………………………………………………………….10

Supplemental Panel 2. Additional details of machine learning models…………………………………………………….14

Supplemental Table 6. Additional details of machine learning models………………………………………………….15

Supplemental Table 7. TRIPOD checklist……………………………………………………………………………………………16

Supplemental Figure 3. Comparison of iScore and THRIVE scoring system for predicting three-year composite outcome…………………………………………………………………………………………………..18

Supplemental Table 8. Comparison of iScore and THRIVE scoring systems for predicting 3-year composite outcome…………………………………………………………………………………………………..18

Supplemental Figure 4. ROC curves for iScore and THRIVE score for predicting 1-year composite outcome (left), and 1-year mortality (right). The curves are almost overlapping each other with no statistically significant difference assessed by DeLong test…………………………………………………………………………………………………19

Supplemental Table 9. Comparison of iScore and THRIVE scoring systems for predicting 1-year outcomes……….19

Supplemental Figure 5. External validation of iScore and THRIVE scores for predicting 3-year composite outcome in validation cohort of 30% of study population……………….............20

Supplemental Table 10. Results of iScore and THRIVE scoring system:3-year prediction of composite outcomes in validation cohort of 30% of study population……………………….................20

Supplemental Figure 6. ROC curves for iScore and THRIVE scores for predicting a 90-day composite outcome…..21

Supplemental Table 11. Comparison of iScore and THRIVE scores for predicting a 90-day composite outcome….21

Supplemental Table 12. Sensitivity and specificity values of the machine learning models for predicting The outcomes…………………………………………………………………………………………………...22

References………………………………………………………………………………………………………...............................23

Panel 1. Acute ischemic stroke*: International Classification of Disease Clinical Modification Tenth Revision*

I63.00, I63.011, I63.012, I63.013, I63.02, I63.031, I63.033, I63.039, I63.09, I63.10, I63.111, I63.112, I63.113, 163.119, I63.12, I63.131, I63.132, I63.133, I63.19, I63.20, I63.211, I63.213, I63.219, I63.22, I63.231, I63.232, I63.233, I63.139, I63.29, I63.30, I63.311, I63.313, I63.319, I63.319, I63.321, I63.323, I63.329, I63.331, I63.339, I63.341, I63.342, I63.349, I63.39, I63.40, I63.411, I63.412, I63.413, I63.421, I63.422, I63.423, I63.429, I63.432, I63.433, I63.439, I63.441, I63.442, I63.449, I63.49, I63.50, I63.511, I63.512, I63.513, I63.519, I63.521, I63.522, I63.523, I63.529, I63.532, I63.533,I63.539, I63.541, I63.542, I63.543, I63.549, I63.59, I63.6, I63.8, I63.81, I63.81, I63.89, I63.9

Supplemental Table 1. Full set of variables from multiple domains for training of machine learning models

Demographics
Age, sex, race
**Social determinants**
Cigarette smoking, substance use
**Anthropometric measures**
**Body mass index**
**Comorbidities**
Anemia, arthritis, atrial fibrillation, asthma, coronary artery disease, cancer, chronic kidney disease, chronic obstructive pulmonary disease, depression, dementia, diabetes mellitus, heart failure, hyperlipidemia, hypertension, hypothyroidism, liver disease, migraine, obstructive sleep apnea, Parkinson disease, peripheral artery disease, seizures, valve disease
**Stroke- specific characteristics**
Anterior circulation stroke, large artery atherosclerosis stroke, cardioembolism-related stroke, small vessel occlusion-related stroke, stroke of undetermined etiology, stroke of determined etiology, National Institute of Health Stroke Scale score, hemiparesis, hemisensory. defect, hemineglect, impaired speech, hemianopia, gaze palsy, facial palsy, arm weakness, lower extremity weakness, dysarthria, ataxia, gait imbalance, vertigo, coma, visual symptoms, incoordination, headache, and confusion
**Vital signs**
Systolic blood pressure, diastolic blood pressure
**Basic activities of daily living**
Dressing-independent (1), dressing-dependent (0), dressing-assisted (2); grooming-independent (1), grooming -dependent (0), grooming -assisted (2); toileting -independent (1), toileting -dependent (0), toileting -assisted (2); eating-independent (1), eating-dependent (0), eating-assisted (2); hygiene-independent (1), hygiene -dependent (0), hygiene -assisted (2); bathing-independent (1), bathing-dependent (0), bathing-assisted (2); mobility-independent (1), mobility-dependent (0), mobility-assisted (2).
**Therapeutic interventions**
Thrombolytics, embolectomy, aspirin, clopidogrel, Aggrenox, warfarin, direct acting oral anticoagulants, statin, high-intensity statin, angiotensin-converting enzyme inhibitor/angiotensin receptor blocker, beta blocker, calcium channel blocker, hydrochlorothiazide, oral diabetic medication, insulin, selective serotonin receptor inhibitor, antiepileptics, antiplatelet, all anticoagulants, antiplatelet, any antihypertensive medications

Supplemental Table 2. Description of machine learning models

Model	Description	Advantageous	limitations
**Extreme Gradient Boost Machine (XGBOOST)**	An ensemble decision tree-based gradient boosting supervised algorithm for classification and regression of prediction models. It is fast and capable of parallel computation and cross-validation to identify important variables.	It provides high predictive accuracy, flexibility, and capability of hyperparameter tuning.	Sensitive to outlier and does not perform well on unstructured data
**Random Forest (RF)**	A supervised machine learning model generated from decision tree algorithms used for regression and classification	Improved performance over decision tree algorithm, capable of handling missing data, produce prediction output without hyperparameter tuning	Long computational time and ineffective for real- time predictions
**Support vector machine (SVM)**	A supervised machine learning model for classification and regression analysis	Performs well with high-dimensional, unstructured data with clear margin of separation between classes and lower risk of over-fitting	Not suitable for large dataset with overlapping classes. Difficult fine- tuning of hyper-parameters.

Supplemental Table 3. Description iScore and THRIVE risk prediction tools.

Risk prediction score	Description
**iScore**	A point-based risk prediction model developed using logistic regression analysis to predict death at 30 days and 1 year in large cohort of patients with acute ischemic stroke (n=12,262) from the Canadian Stroke Network Registry [1]. Based on age, age, sex, stroke severity assessed with the Canadian Neurological Scale, stroke subtype according to the Trial of ORG 10172 in Acute Stroke Treatment (TOAST) 2, glucose level, comorbid atrial fibrillation, congestive heart failure, cancer, kidney disease, and preadmission dependency an integer score is calculated with higher the score greater the mortality. The original scoring system had a c-statistic of 0.85 at 30 days and 0.82 at 1 year in development set (n=8,223) and 0.85 at 30 days and 0.84 in internal validation set (n=4,039) and 0.79 at 30 days and 0.78 at 1 year in external validation cohort (n=3,272 from Ontario Stroke Audit). The iScore was subsequently externally validated in several studies 1,3-14 and available as online calculator 15.
**THRIVE**	A 5- predictor variable (age, stroke severity, and history of hypertension, diabetes mellitus and atrial fibrillation) point-based risk prediction tool developed using logistic regression analysis to predict functional outcome at 3-months in a cohort of patients (n=305) receiving endovascular treatment for ischemic stroke 16. The score ranged from 0-9 with higher the score poor the outcome. The THRIVE scoring system was subsequently externally validated in several studies 5-7,16-24.

Abbreviations: iScore, Ischemic Stroke Predict to Risk Score; THRIVE, Totaled Health Risks in Vascular Events

Supplemental Table 4. Original iScore and THRIVE point-based prediction scores for validation and recalibration in the study cohort.

Variables	Components	Number of points		
		Points	30-day score	1-year score
**iScore**				
**Age**			+ Age in years	+ Age in years
**Sex**	Female			
	Male		+10	+5
**Stroke severity**	0		0	0
	≤ 4		+40	+25
	5 – 7		+65	+40
	≥ 8		+105	+40
**Stroke type**	Lacunar		0	0
	Non-lacunar		+30	+15
	Undetermined		+35	+20
**Risk factors**	Atrial fibrillation		+10	+5
	CHF		+10	+10
	Previous myocardial infarction			+5
	Current smoker			+5
**Comorbid conditions**	Cancer		+10	+15
	Renal dialysis		+35	+40
**Pre-admission disability**	Independent		0	0
	dependent		+20	+20
**Glucose on admission**	<135 mg/dL		0	0
	<135 mg/dL		+15	+20
**THRIVE**				
**Age, years**	≤ 59	0		
	60 – 79	1		
	≥ 80	2		
**Chronic disease**				
	0 (HTN, DM, or atrial fibrillation)	0		
	1 (HTN, DM, or atrial fibrillation)	1		
	2 (HTN, DM, or atrial fibrillation)	2		
	3 (HTN, DM, or atrial fibrillation)	3		
**NIHSS score**	≤ 10	0		
	11 – 20	2		
	≥ 21	4		
**Total score**		9		

Abbreviations: DM, diabetes mellitus; HTN, hypertension; iScore, Ischemic Stroke Predict to Risk Score; NIHSS, National Institute of Health Stroke Scale THRIVE, Totaled Health Risks in Vascular Events

Supplemental Figure 1. Strengthening The Reporting of Observational studies in Epidemiology (STROBE) flow diagram of final cohort selection

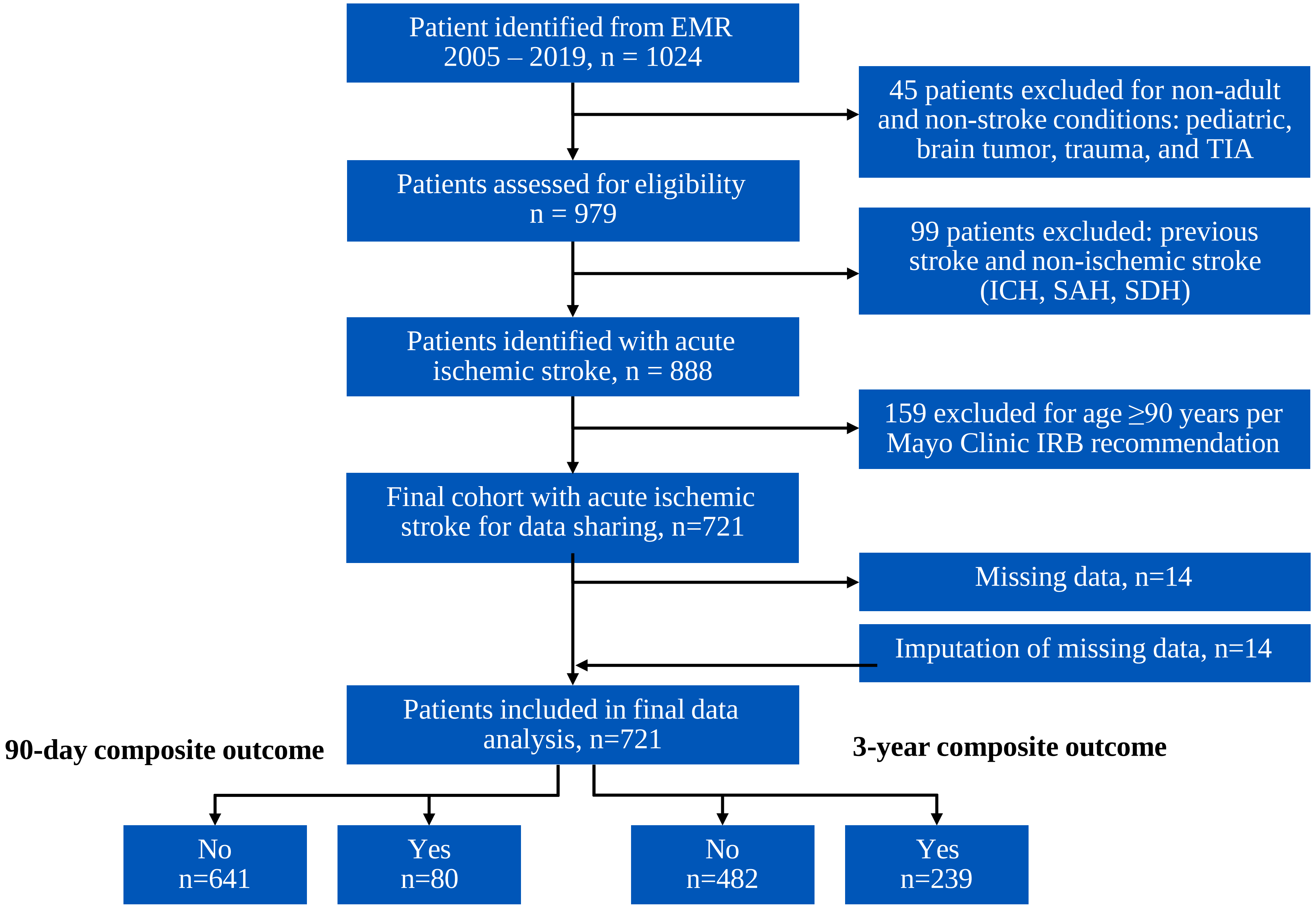

Supplemental Figure 2. Schematic representation of the process of the development of machine learning algorithms: datasets, methods of development, and cross-validation.

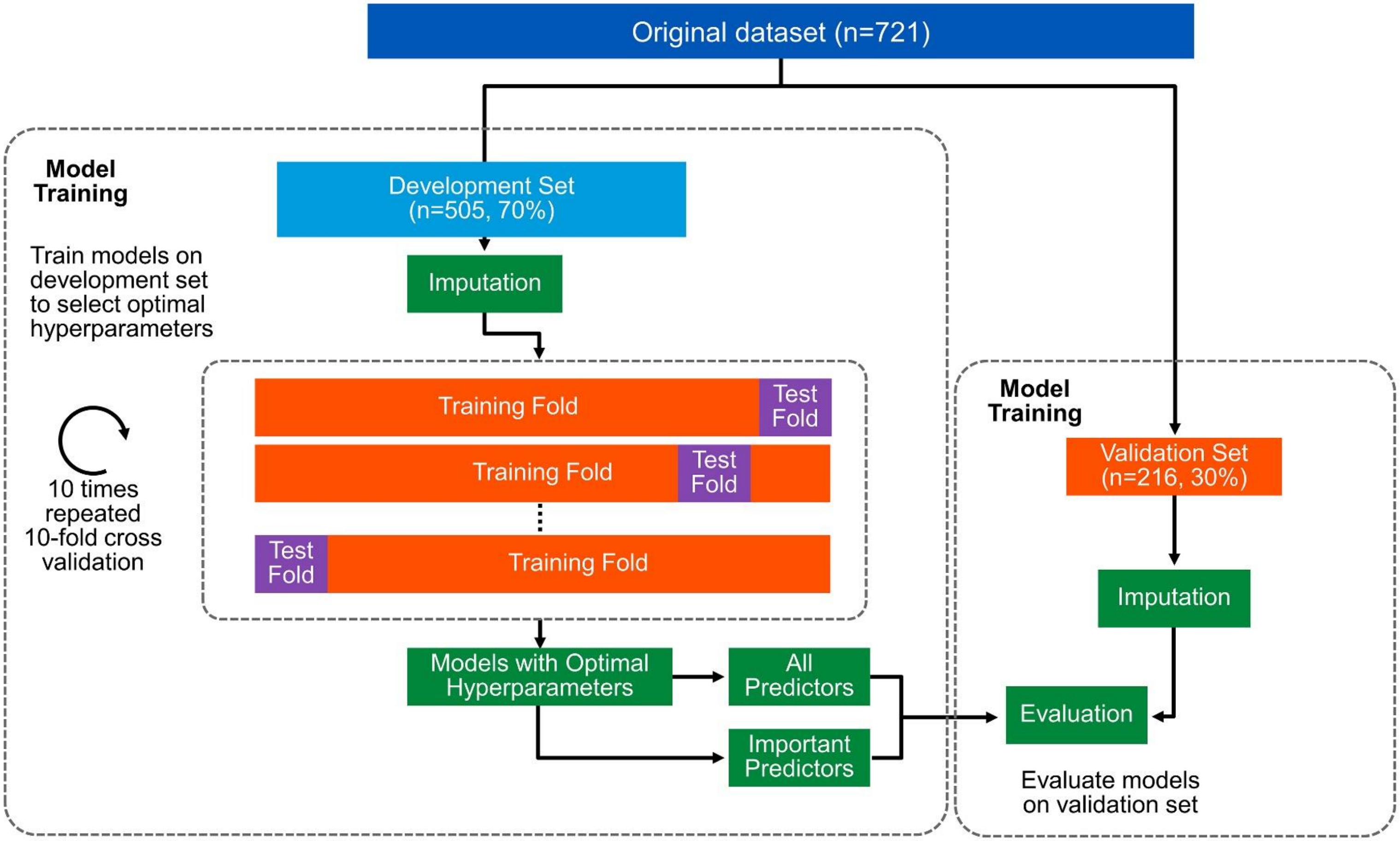

The flow diagram shows the process of data splitting (70:30 into development and validation sets), imputation of missing data (missForest with package R), hyperparameter tuning, cross validation (10 times repeated and 10-fold cross validation), and sequential validation. Additionally, the figure showcases the process of model fitting, where each machine learning algorithm is fit using cross-validation. The best performing model for

each algorithm, with optimal hyperparameters, is subsequently selected.

Supplemental Table 5. Strengthening The Reporting of Observational studies in Epidemiology (STROBE) check list

Section/Topic	Item No	Recommendation	Reported on Page No
Title and abstract	1	(*a*) Indicate the study's design with a commonly used term in the title or the abstract	1
		(*b*) Provide in the abstract an informative and balanced summary of what was done and what was found	3
Introduction			
Background/rationale	2	Explain the scientific background and rationale for the investigation being reported	5, 6
Objectives	3	State specific objectives, including any pre-specified hypotheses	6
Methods			
Study design	4	Present key elements of study design early in the paper	6,7
Setting	5	Describe the setting, locations, and relevant dates, including periods of recruitment, exposure, follow-up, and data collection	6,7
Participants	6	(*a*) *Cohort study*—Give the eligibility criteria, and the sources and methods of selection of participants. Describe methods of follow-up<br>*Case-control study*—Give the eligibility criteria, and the sources and methods of case ascertainment and control selection. Give the rationale for the choice of cases and controls.<br>*Cross-sectional study*—Give the eligibility criteria, and the sources and methods of selection of participants.	6, 7
		(*b*) *Cohort study*—For matched studies, give matching criteria and number of exposed and unexposed<br>*Case-control study*—For matched studies, give matching criteria and the number of controls per case.	
Variables	7	Clearly define all outcomes, exposures, predictors, potential confounders, and effect modifiers. Give diagnostic criteria, if applicable	7
Data sources/measurement	8*	For each variable of interest, give sources of data and details of methods of assessment (measurement). Describe comparability of assessment methods if there is more than one group.	6-10
Bias	9	Describe any efforts to address potential sources of bias	6-10
Study size	10	Explain how the study size was arrived at	Not applicable

Quantitative

variables	11	Explain how quantitative variables were handled in the analyses. If applicable, describe which groupings were chosen and why	6-10

Statistical methods	12	(*a*) Describe all statistical methods, including those used to control for confounding	10, 11
		(*b*) Describe any methods used to examine subgroups and interactions	
		(*c*) Explain how missing data were addressed	7, 8
		(*d*) *Cohort study*—If applicable, explain how loss to follow-up was addressed<br>*Case-control study*—If applicable, explain how matching of cases and controls was addressed.<br>*Cross-sectional study*—If applicable, describe analytical methods taking account of sampling strategy	N/A
		(*e*) Describe any sensitivity analyses	N/A

Results			
Participants	13*	(a) Report numbers of individuals at each stage of study—e.g., numbers potentially eligible, examined for eligibility, confirmed eligible, included in the study, completing follow-up, and analysed	12-14
		(b) Give reasons for non-participation at each stage	
		(c) Consider use of a flow diagram	
Descriptive data	14*	(a) Give characteristics of study participants (e.g., demographic, clinical, social) and information on exposures and potential confounders	Table 1, 7
		(b) Indicate number of participants with missing data for each variable of interest	7, 8
		(c) *Cohort study*—Summarize follow-up time (e.g., average, and total amount)	8
Outcome data	15*	*Cohort study*—Report numbers of outcome events or summary measures over time	8
		Case-control study—Report numbers in each exposure category, or summary measures of exposure	8
		Cross-sectional study—Report numbers of outcome events or summary measures	8
Main results	16	(*a*) Give unadjusted estimates and, if applicable, confounder-adjusted estimates and their precision (e.g., 95% confidence interval). Make clear which confounders were adjusted for and why they were included	7
		(*b*) Report category boundaries when continuous variables were categorized	7
		(*c*) If relevant, consider translating estimates of relative risk into absolute risk for a meaningful time period	
Other analyses	17	Report other analyses done—e.g., analyses of subgroups and interactions, and sensitivity analyses	N/A
Discussion			
Key results	18	Summarize key results with reference to study objectives	12-14
Limitations	19	Discuss limitations of the study, taking into account sources of potential bias or imprecision. Discuss both direction and magnitude of any potential bias	18

Interpretation	20	Give a cautious overall interpretation of results considering objectives, limitations, multiplicity of analyses, results from similar studies, and other relevant evidence	18, 19
Generalizability	21	Discuss the generalizability (external validity) of the study results	18
Other Information			
Funding	22	Give the source of funding and the role of the funders for the present study and, if applicable, for the original study on which the present article is based	11, 12

**Give information separately for cases and controls in case-control studies and, if applicable, for exposed and unexposed groups in cohort and cross-sectional studies.*

Note: An Explanation and Elaboration article discusses each checklist item and gives methodological background and published examples of transparent reporting. The STROBE checklist is best used in conjunction with this article (freely available on the Web sites of PLoS Medicine at http://www.plosmed

Panel 2. Additional details of machine learning models

Data variables
The two primary outcome variables were the occurrence of recurrent stroke or mortality within 90-days and 3-years of hospital discharge among patients with AIS. A total of 90 independent variables were recorded for each patient, including demographics, length of hospital stays, social indicators, severity of stroke assessed by National Institute of Health Stroke Scale (NIHSS), stroke subtypes according to Trial of Org 10172 in Acute Stroke Treatment (TOAST) classification and comorbidities [2]. Table 1 reports the recorded patients' data for 90-day and 3-year outcomes, respectively. Comorbidities included the presence of hypertension, dyslipidemia, depression, heart failure, atrial fibrillation, coronary artery disease, peripheral artery disease, chronic obstructive pulmonary disease, chronic kidney disease, cancer, dementia, obstructive sleep apnea, obesity or osteoarthritis before initial admission. Vitals and pathology data included systolic and diastolic blood pressure (average of three consecutive systolic and three consecutive diastolic blood pressures on admission), heart rate (average of three heart rates on admission), blood glucose, blood urea nitrogen, creatinine,
and hemoglobin levels, medications on dismissal, discharge disposition, and activities of Daily Living.

Data processing and sub sampling for class imbalance
A total of 14 (1.9%) patients contained one or more missing data. Imputation of missing values was performed using random forest imputation with the missForest package in R. Imputation was performed independently on training and test sets to prevent data leakage. A complete list of the variables along with their descriptive statistics is provided in Table 1. Baseline characteristics and recurrent stroke or mortality within 90-days and 3-years of discharge status of the cohorts were assessed. For continuous variables, the mean (SD) for sufficiently normally distributed variables and median (IQR) for non-normally distributed variables were reported. For categorical variables, the number (proportion) is reported. Comparisons between the groups for continuous variables were conducted with one-way analysis of variance (ANOVA) test for sufficiently normally distributed data, and Kruskal-Walli's test for non-normally distributed data. Comparisons between the groups for categorical variables was conducted with Pearson χ^2 test. Differences between the groups were deemed statistically significant at a p-value of 5% or lower.

Model development
The data was randomly partitioned with a split of 70/30 into development and validation sets, respectively. To develop the models to predict 90-day and 3-year recurrent stroke or mortality, we employed ten times repeated ten-fold cross-validation using the development set. Evaluation of the models was performed on the validation set. Figure 2 illustrates the overall model development and evaluation process. The statistical models were based on logistic regression (LR) and least absolute shrinkage and selection operator (LASSO) logistic regression. The machine learning-based models included random forest (RF), support vector machine (SVM), and extreme gradient boosting decision trees (XGBOOST). The full set of variables were used as input for each of the models. Hyperparameter tuning of models was performed by a grid search of values assessed for each model hyperparameter during the cross-validation on the development set. Optimal hyperparameters were selected based on the minimum Brier score across the cross-validation, and the final model was fit to the development set with the optimal hyperparameters. To compare overall performance of the models, we calculated the area under the receiver operating characteristic curve (AUC), sensitivity, specificity, positive predictive value (PPV) and model training time in seconds. 95% confidence intervals for AUC were calculated via bootstrap resampling in 2000 resamples. We reported the performance metrics at the default 0.5 probability threshold. Data was standardized prior to model training and validation. One variable ("psychiatric") had zero variance and was removed during model development, due to lack of predictive ability. Model development were performed using the Caret package in RStudio [10] on computer with a Ryzen 7 3700X 8-Core 4.2GHz processor.

Supplemental Table 6. Additional details of machine learning models

Model	Hyperparameters	Value(s)	Final 90-day value	Final 3-year value
**RF**	Number of variables randomly sampled as candidates at each split	1. [2, 50, 98] (default)	50	2
**SVM**	Sigma	0.005934468* (default)	0.005934468	0.005934468
	C	[0.25, 0.5, 1]	1	1
	Kernel	Radial basis kernel	Radial basis kernel	Radial basis kernel
**XGBOOST**	Number of rounds (nrounds)	[50, 100] (default)	50	100
	Maximum tree depth (max_depth)	[1, 2] (default)	2	2
	Learning rate (eta)	[0.1, 0.3] (default)	0.1	0.1
	Gamma	2. 0 (default)	3. 0	4. 0
	Column sampling (colsample_bytree)	5. [0.8, 1.0] (default)	6. 0.8	7. 0.8
	Minimum leaf weight (min_child_weight)	8. 1 (default)	9. 1	10. 1
	Row sampling (subsample)	11. 1 (default)	12. 1	13. 3
**14. * SVM default sigma value calculated automatically using the kernlab package, estimating the optimal value based on the data using the sigest() function, which is based upon the median (0.5 quantile) of |x-x'|^2.**				

Supplemental Table 7. TRIPOD checklist

Section/Topic	Item		Checklist Item	Page
**Title and abstract**				
Title	1	D; V	Identify the study as developing and/or validating a multivariable prediction model, the target population, and the outcome to be predicted.	1
Abstract	2	D;V	Provide a summary of objectives, study design, setting, participants, sample size, predictors, outcome, statistical analysis, results, and conclusions.	2
**Introduction**				
Background and objectives	3a	D;V	Explain the medical context (including whether diagnostic or prognostic) and rationale for developing or validating the multivariable prediction model, including references to existing models.	5 - 6
	3b	D;V	Specify the objectives, including whether the study describes the development or validation of the model or both.	6
**Methods**				
Source of data	4a	D;V	Describe the study design or source of data (e.g., randomized trial, cohort, or registry data), separately for the development and validation data sets, if applicable.	6-7
	4b	D;V	Specify the key study dates, including start of accrual; end of accrual; and, if applicable, end of follow-up.	6-7
Participants	5a	D;V	Specify key elements of the study setting (e.g., primary care, secondary care, general population) including number and location of centres.	6-7
	5b	D;V	Describe eligibility criteria for participants.	7
	5c	D;V	Give details of treatments received, if relevant.	N/A
Outcome	6a	D;V	Clearly define the outcome that is predicted by the prediction model, including how and when assessed.	9
	6b	D;V	Report any actions to blind assessment of the outcome to be predicted.	N/A
Predictors	7a	D;V	Clearly define all predictors used in developing or validating the multivariable prediction model, including how and when they were measured.	7 -8
	7b	D;V	Report any actions to blind assessment of predictors for the outcome and other predictors.	7-8
Sample size	8	D;V	Explain how the study size was arrived at.	6-10
Missing data	9	D;V	Describe how missing data were handled (e.g., complete-case analysis, single imputation, multiple imputation) with details of any imputation method.	7
Statistical analysis methods	10a	D	Describe how predictors were handled in the analyses.	7-9
	10b	D	Specify type of model, all model-building procedures (including any predictor selection), and method for internal validation.	8
	10c	V	For validation, describe how the predictions were calculated.	7-8
	10d	D;V	Specify all measures used to assess model performance and, if relevant, to compare multiple models.	9-10
	10e	V	Describe any model updating (e.g., recalibration) arising from the validation, if done.	9
Risk groups	11	D;V	Provide details on how risk groups were created, if done.	N/A
Development vs. validation	12	V	For validation, identify any differences from the development data in setting, eligibility criteria, outcome, and predictors.	10-11
**Results**				
Participants	13a	D;V	Describe the flow of participants through the study, including the number of participants with and without the outcome and, if applicable, a summary of the follow-up time. A diagram may be helpful.	Suppl 4
	13b	D;V	Describe the characteristics of the participants (basic demographics, clinical features, available predictors), including the number of participants with missing data for predictors and outcome.	Suppl 13-16
	13c	V	For validation, show a comparison with the development data of the distribution of important variables (demographics, predictors and outcome).	Figure 2-4
Model development	14a	D	Specify the number of participants and outcome events in each analysis.	7
	14b	D	If done, report the unadjusted association between each candidate predictor and outcome.	N/A
Model specification	15a	D	Present the full prediction model to allow predictions for individuals (i.e., all regression coefficients, and model intercept or baseline survival at a given time point).	Figure 2-4
	15b	D	Explain how to the use the prediction model.	Table 1
Model performance	16	D;V	Report performance measures (with CIs) for the prediction model.	Table 1, Figures 2-4
Model-updating	17	V	If done, report the results from any model updating (i.e., model specification, model performance).	N/A
**Discussion**				
Limitations	18	D;V	Discuss any limitations of the study (such as nonrepresentative sample, few events per predictor, missing data).	16

Interpretation	19a	V	For validation, discuss the results with reference to performance in the development data, and any other validation data.	13
	19b	D;V	Give an overall interpretation of the results, considering objectives, limitations, results from similar studies, and other relevant evidence.	13-14
Implications	20	D;V	Discuss the potential clinical use of the model and implications for future research.	15
**Other information**				
Supplementary information	21	D;V	Provide information about the availability of supplementary resources, such as study protocol, Web calculator, and data sets.	6
Funding	22	D;V	Give the source of funding and the role of the funders for the present study.	3

*Items relevant only to the development of a prediction model are denoted by D, items relating solely to a validation of a prediction model are denoted by V, and items relating to both are denoted D;V.We recommend using the TRIPOD Checklist in conjunction with the TRIPOD Explanation and Elaboration doc

Supplemental Figure 3. Comparison of iScore and THRIVE scoring system for predicting three-year composite outcome.

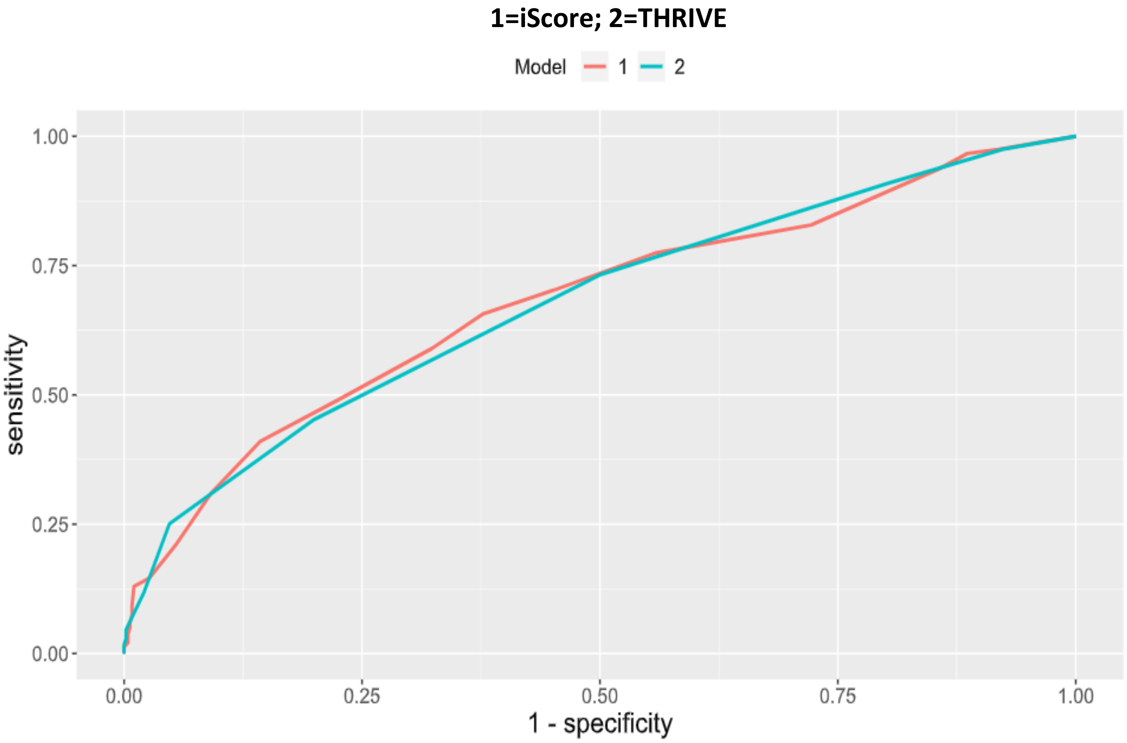

Table 8. Comparison of iScore and THRIVE scoring systems for predicting 3-year composite outcome.

Outcome	iScore		THRIVE		DeLong test, P=
	AUC	CI	AUC	CI	
3-year composite	0.6788	0.6361-0.7215	0.6756	0.6342-0.7171	0.8856

Supplemental Figure 4. ROC curves for iScore and THRIVE score for predicting 1-year composite outcome (left), and 1-year mortality (right). The curves are almost overlapping each other with no statistically significant difference assessed by De Long test.

1=iScore; 2=THRIVE

1-year prediction of composite outcome 1-year prediction of mortality

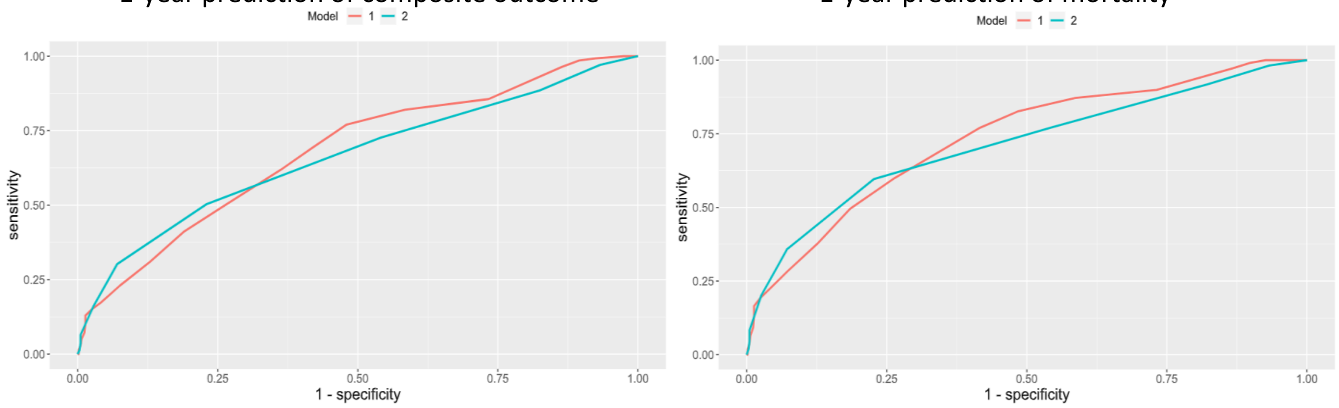

Supplemental Table 9. Comparison of iScore and THRIVE scoring systems for predicting 1-year outcomes.

Outcome	iScore		THRIVE		DeLong test
	AUC	CI	AUC	CI	
**1 year composite**	0.6801	0.6306-0.7296	0.6631	0.6094-0.7168	0.4943
**1 year mortality**	0.7335	0.6823-0.7848	0.7132	0.6561-0.7704	0.4320

Supplemental Figure 5. External validation of iScore and THRIVE scores for predicting 3-year composite outcome in validation cohort of 30% of study population.

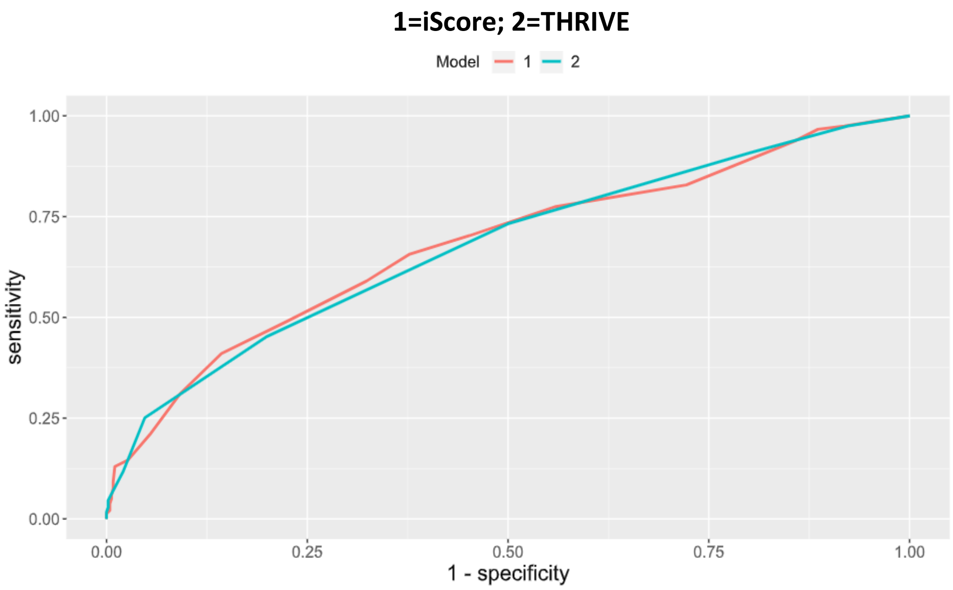

Supplemental Table 10. Results of iScore and THRIVE scoring system:3-year prediction of composite outcome in validation cohort of 30% of study population.

**Outcome**	**iScore**		**THRIVE**		**DeLong test**
	AUC	CI	AUC	CI	
**3-year composite**	0.6788	0.6361-0.7215	0.6756	0.6342-0.7171	0.8856

Figure 6. ROC curves for iScore and THRIVE scores for predicting a 90-day composite outcome.

1=iScore; 2=THRIVE

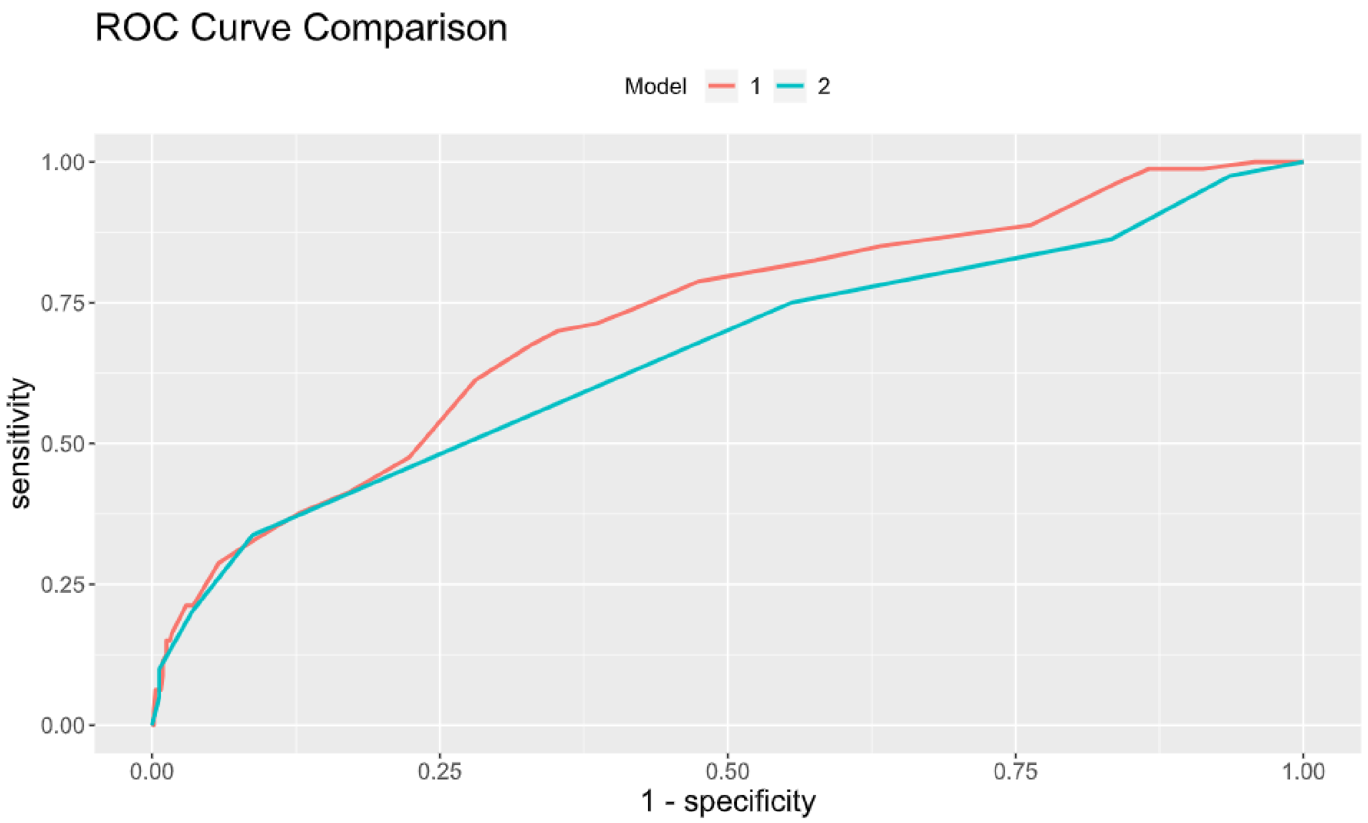

Table 11. Comparison of iScore and THRIVE scores for predicting a 90-day composite outcome.

Outcome	iScore		THRIVE		DeLong test, P=
	AUC	CI	AUC	CI	
**90-day composite**	0.7152	0.6536-0.7768	0.6548	0.5848-0.7248	0.0233

Supplemental Table 12. Sensitivity and specificity values of machine learning models for predicting the outcomes

Prediction	Variables	Model	Sensitivity	specificity
**90-day prediction of composite outcome**	All 90 variables	RF	0.15	0.99
		SVM	0.15	1.00
		XGBOOST	0.15	0.99
	Top 10 identified variables	RF	0.15	1.00
		SVM	0.15	1.00
		XGBOOST	0.20	1.00
**3-year prediction of composite outcome**	All 90 variables	RF	0.22	0.82
		SVM	0.38	0.89
		XGBOOST	0.45	0.89
	Top 10 identified variables	RF	0.35	0.88
		SVM	0.42	0.88
		XGBOOST	0.43	0.89

References

1. Saposnik G, Kapral MK, Liu Y, Hall R, O'Donnell M, Raptis S, Tu JV, Mamdani M, Austin PC. IScore: a risk score to predict death early after hospitalization for an acute ischemic stroke. *Circulation*. 2011;123:739-749. doi: 10.1161/circulationaha.110.983353
2. Adams HP, Jr., Bendixen BH, Kappelle LJ, Biller J, Love BB, Gordon DL, Marsh EE, 3rd. Classification of subtype of acute ischemic stroke. Definitions for use in a multicenter clinical trial. TOAST. Trial of Org 10172 in Acute Stroke Treatment. *Stroke*. 1993;24:35-41.
3. Béjot Y, Daubail B, Sensenbrenner B, Legris N, Durier J, Giroud M. iScore for predicting institutional care after ischemic stroke: a population-based study. *J Stroke Cerebrovasc Dis*. 2015;24:694-698. doi: 10.1016/j.jstrokecerebrovasdis.2014.11.010
4. Béjot Y, Jacquin A, Daubail B, Durier J, Giroud M. Population-based validation of the iScore for predicting mortality and early functional outcome in ischemic stroke patients. *Neuroepidemiology*. 2013;41:169-173. doi: 10.1159/000354634
5. Dragoumanos V, Tzirogiannis KN, Panoutsopoulos GI, Krikonis K, Fousteris E, Vourvou M, Elesnitsalis G, Melas N, Kourentzi KT, Melidonis A. Evaluation of IScore validity in a Greek cohort of patients with type 2 diabetes. *BMC Neurol*. 2013;13:121. doi: 10.1186/1471-2377-13-121
6. Ewara EM, Isaranuwatchai W, Bravata DM, Williams LS, Fang J, Hoch JS, Saposnik G. The iScore predicts total healthcare costs early after hospitalization for an acute ischemic stroke. *Int J Stroke*. 2015;10:1179-1186. doi: 10.1111/ijs.12641
7. Nikneshan D, Raptis R, Pongmoragot J, Zhou L, Johnston SC, Saposnik G. Predicting clinical outcomes and response to thrombolysis in acute stroke patients with diabetes. *Diabetes Care*. 2013;36:2041-2047. doi: 10.2337/dc12-2095
8. Saposnik G, Demchuk A, Tu JV, Johnston SC. The iScore predicts efficacy and risk of bleeding in the National Institute of Neurological disorders and Stroke Tissue Plasminogen Activator Stroke Trial. *J Stroke Cerebrovasc Dis*. 2013;22:876-882. doi: 10.1016/j.jstrokecerebrovasdis.2012.09.001
9. Saposnik G, Fang J, Kapral MK, Tu JV, Mamdani M, Austin P, Johnston SC. The iScore predicts effectiveness of thrombolytic therapy for acute ischemic stroke. *Stroke*. 2012;43:1315-1322. doi: 10.1161/strokeaha.111.646265
10. Saposnik G, Raptis S, Kapral MK, Liu Y, Tu JV, Mamdani M, Austin PC. The iScore predicts poor functional outcomes early after hospitalization for an acute ischemic stroke. *Stroke*. 2011;42:3421-3428. doi: 10.1161/strokeaha.111.623116
11. Saposnik G, Reeves MJ, Johnston SC, Bath PM, Ovbiagele B. Predicting clinical outcomes after thrombolysis using the iScore: results from the Virtual International Stroke Trials Archive. *Stroke*. 2013;44:2755-2759. doi: 10.1161/strokeaha.113.001343
12. Van Hooff RJ, Nieboer K, De Smedt A, Moens M, De Deyn PP, De Keyser J, Brouns R. Validation assessment of risk tools to predict outcome after thrombolytic therapy for acute ischemic stroke. *Clin Neurol Neurosurg*. 2014;125:189-193. doi: 10.1016/j.clineuro.2014.08.011
13. Wang WY, Sang WW, Jin D, Yan SM, Hong Y, Zhang H, Yang X. The Prognostic Value of the iScore, the PLAN Score, and the ASTRAL Score in Acute Ischemic Stroke. *J Stroke Cerebrovasc Dis*. 2017;26:1233-1238. doi: 10.1016/j.jstrokecerebrovasdis.2017.01.013

14. Zhang N, Liu G, Zhang G, Fang J, Wang Y, Zhao X, Pan Y, Guo L, Wang Y. External validation of the iScore for predicting ischemic stroke mortality in patients in China. *Stroke*. 2013;44:1924-1929. doi: 10.1161/strokeaha.111.000172
15. Score IISPR. SORCan. http://www.sorcan.ca/iscore/index.html. 2010. Accessed September 21, 2022.
16. Flint AC, Cullen SP, Faigeles BS, Rao VA. Predicting long-term outcome after endovascular stroke treatment: the totaled health risks in vascular events score. *AJNR Am J Neuroradiol*. 2010;31:1192-1196. doi: 10.3174/ajnr.A2050
17. Boehme AK, Rawal PV, Lyerly MJ, Albright KC, Bavarsad Shahripour R, Palazzo P, Kapoor N, Alvi M, Houston JT, Harrigan MR, et al. Investigating the utility of previously developed prediction scores in acute ischemic stroke patients in the stroke belt. *J Stroke Cerebrovasc Dis*. 2014;23:2001-2006. doi: 10.1016/j.jstrokecerebrovasdis.2014.02.003
18. Chen W, Liu G, Fang J, Wang Y, Song Y, Pan Y, Li H, Liu L, Wang C, Wang DZ, et al. External Validation of the Totaled Health Risks in Vascular Events Score to Predict Functional Outcome and Mortality in Patients Entered into the China National Stroke Registry. *J Stroke Cerebrovasc Dis*. 2016;25:2331-2337. doi: 10.1016/j.jstrokecerebrovasdis.2016.03.021
19. Chen W, Pan Y, Zhao X, Liao X, Liu L, Wang C, Wang Y, Wang Y. Totaled health risks in vascular events score predicts clinical outcome and symptomatic intracranial hemorrhage in chinese patients after thrombolysis. *Stroke*. 2015;46:864-866. doi: 10.1161/strokeaha.114.007979
20. Flint AC, Cullen SP, Rao VA, Faigeles BS, Pereira VM, Levy EI, Jovin TG, Liebeskind DS, Nogueira RG, Jahan R, et al. The THRIVE score strongly predicts outcomes in patients treated with the Solitaire device in the SWIFT and STAR trials. *Int J Stroke*. 2014;9:698-704. doi: 10.1111/ijs.12292
21. Flint AC, Xiang B, Gupta R, Nogueira RG, Lutsep HL, Jovin TG, Albers GW, Liebeskind DS, Sanossian N, Smith WS. THRIVE score predicts outcomes with a third-generation endovascular stroke treatment device in the TREVO-2 trial. *Stroke*. 2013;44:3370-3375. doi: 10.1161/strokeaha.113.002796
22. Kamel H, Patel N, Rao VA, Cullen SP, Faigeles BS, Smith WS, Flint AC. The totaled health risks in vascular events (THRIVE) score predicts ischemic stroke outcomes independent of thrombolytic therapy in the NINDS tPA trial. *J Stroke Cerebrovasc Dis*. 2013;22:1111-1116. doi: 10.1016/j.jstrokecerebrovasdis.2012.08.017
23. Kastrup A, Brunner F, Hildebrandt H, Roth C, Winterhalter M, Gießing C, Papanagiotou P. THRIVE score predicts clinical and radiological outcome after endovascular therapy or thrombolysis in patients with anterior circulation stroke in everyday clinical practice. *Eur J Neurol*. 2017;24:1032-1039. doi: 10.1111/ene.13328
24. Lei C, Wu B, Liu M, Chen Y, Yang H, Wang D, Lin S, Hao Z. Totaled health risks in vascular events score predicts clinical outcomes in patients with cardioembolic and other subtypes of ischemic stroke. *Stroke*. 2014;45:1689-1694. doi: 10.1161/strokeaha.113.004352
